# Understanding healthcare accessibility for military veterans living with Charles Bonnet Syndrome: co-production of a psychoeducational toolkit

**DOI:** 10.3389/fpsyg.2026.1698432

**Published:** 2026-06-03

**Authors:** Jemma McCready, Renata S. M. Gomes, Matthew D. Kiernan, Jane Arnfield, Emma Senior, Derek P. Farrell

**Affiliations:** 1 Department of Psychology, Faculty of Health and Life Sciences, Northumbria University, Newcastle upon Tyne, United Kingdom; 2 Northern Hub for Veteran and Military Families’ Research, Northumbria University, Newcastle upon Tyne, United Kingdom; 3 NHS Blood and Transplant, National Health Service, Bristol, United Kingdom; 4 Faculty of Society and Culture, Northumbria University, Newcastle upon Tyne, United Kingdom; 5 Department of Nursing, Midwifery & Health, Faculty of Health & Life Sciences, Northumbria University, Newcastle Upon Tyne, United Kingdom; 6 School of Nursing & Midwifery, Queen’s University, Belfast, Northern Ireland, United Kingdom

**Keywords:** Charles Bonnet Syndrome, co-production, healthcare accessibility, military veterans, psychoeducational resources, visual hallucinations

## Abstract

**Introduction:**

Charles Bonnet Syndrome (CBS) is a condition characterized by visual hallucinations in individuals with visual impairments. Among military veterans, CBS can cause considerable distress, leading to health-related anxiety and delays in seeking help. Despite its prevalence, awareness of CBS remains limited across healthcare services, resulting in underdiagnosis and inadequate support. There is a need to better understand experiences of managing CBS and accessing healthcare services, from both healthcare and service user perspectives. Additionally, tailored resources are needed to address these gaps. This study explored these experiences and co-produced a toolkit to support military veterans, their families, and healthcare professionals in managing CBS and improving access to appropriate care.

**Methods:**

A two-phase, qualitative, co-production design was employed. In Phase 1, military veterans with CBS (*n* = 7) and specialist healthcare professionals working in the visual impairment and veteran sector (*n* = 13) participated in a workshop that explored the current management of CBS, the accessibility of healthcare services, and support needs. Thematic analysis identified key themes that guided the initial development of the toolkit. In Phase 2, a second focus group with military veterans (*n* = 4) and specialist healthcare professionals working in the visual impairment and veteran sector (*n* = 5) gathered feedback on the toolkit and areas for improvement. Thematic analysis was used to identify areas of refinement.

**Results:**

A psychoeducational toolkit was developed in the form of podcasts and a video animation, as preferred by users. One podcast focused on raising awareness of CBS among healthcare professionals and providers, two podcasts focused explicitly on military veterans and their families/carers, and finally, an animated whiteboard video targeted toward all audiences. All the podcasts and video animations included a practical strategy for managing visual hallucinations.

**Conclusion:**

The multiple toolkits developed address key barriers to healthcare, promote early recognition of CBS, and provide accessible self-management strategies for veterans, their families, and healthcare professionals.

## 1 Introduction

### 1.1 Charles Bonnet Syndrome

Charles Bonnet Syndrome (CBS) is a condition in which individuals with complete or partial vision loss experience visual hallucinations that are not attributable to cognitive decline or a psychiatric disorder, and which the individual typically recognizes as unreal (Menon et al., 2003). Hallucinations vary considerably in complexity, ranging from simple shapes, flashes, or colored patterns to detailed images of faces, animals, or moving scenes (Manford and Andermann, 1998), and may last for seconds or persist continuously (Esteves Leandro et al., 2020). Although the precise pathogenesis of CBS remains unclear (Carpenter et al., 2019), the most widely accepted explanation is deafferentation: partial or complete loss of visual input reduces afferent signals to the brain, triggering compensatory hyperactivity in the visual cortex and the spontaneous generation of internally generated images perceived as hallucinations (Ffytche, 2005; Marschall et al., 2020).

CBS is associated with ophthalmological conditions that cause visual impairment, including age-related macular degeneration (ARMD) (Abbott et al., 2007; Niazi et al., 2020), glaucoma (Subhi et al., 2021), and cataracts (Subhi et al., 2022). Global estimates suggest that approximately 239 million individuals have moderate to severe visual impairments, of whom around 20% (∼47.2 million) are expected to experience CBS (Subhi et al., 2022). In the UK alone, approximately one million individuals are currently affected (Potts, 2019), and this figure is projected to rise to four million by 2050 as the population ages (Pezzullo et al., 2018). CBS has been documented across a wide age range and in both sexes, though it most commonly affects adults over 64 years of age (Teunisse et al., 1995), and females appear to be at greater risk than males (Christoph et al., 2025).

### 1.2 CBS in military veteran populations

Military veterans constitute a distinct subset of the population at elevated risk of CBS. It is estimated that over one million veterans in the United States and approximately 59,000 in the UK are living with a visual impairment (Blind Veterans U.K., 2017; Veterans Affairs., 2018), with recent findings suggesting that around 11% of visually impaired veterans experience CBS (Jones et al., 2025b). Demographic projections add further urgency to this picture: by 2028, the UK veteran population is anticipated to include a greater proportion of female veterans (10% in 2016 vs. 13% in 2028) and veterans aged 60–74 (21% vs. 30%) and 90 and over (6% vs. 12%) (Ministry of Defence, 2019). Given that older age and female sex are independently associated with higher CBS rates (Christoph et al., 2025; Teunisse et al., 1995), the number of veterans experiencing CBS-related hallucinations is likely to increase accordingly. Psychosocial risk factors prevalent in veteran communities—including loneliness and social isolation (Jones et al., 2021a)—may further increase susceptibility or worsen the impact of CBS (Christoph et al., 2025).

Despite this, the lived experience of CBS-related visual hallucinations in veterans remains insufficiently studied. Jones et al. (2025a), in a study of 115 military veterans, found that more than three-quarters experienced both simple and complex hallucinations, typically featuring people, animals, and abstract shapes. A small proportion also reported military-related hallucinations involving items or memories from service (Dave et al., 2024), a presentation not commonly reported in the general population. Hallucinations occurred daily or weekly for many veterans, lasting several minutes (63%) or up to an hour (23%), with approximately 40% occurring outside the home—introducing risks to mobility and social participation. Emotional responses were predominantly negative, with fear, confusion, and distress commonly reported, particularly when hallucinations involved threatening content such as screaming faces or mythological creatures (Abdulhussein et al., 2024; Dave et al., 2024; Jones et al., 2025b). Some veterans, however, described feelings of fascination or pleasure in response to vivid or pleasant imagery, suggesting that emotional response is closely tied to hallucination content (Dave et al., 2024). Veterans who perceived hallucinations as bothersome were significantly more likely to have experienced them for over three years and to report more frequent and longer-lasting episodes; newly diagnosed individuals tended to find the experience particularly distressing, suggesting that unfamiliarity heightens the initial impact (Jones et al., 2025b).

### 1.3 CBS in healthcare contexts

Despite its prevalence, awareness of CBS among healthcare professionals remains limited. A survey of 499 Canadian physicians found that 55% were unaware of the condition and 85% did not routinely discuss the possibility of CBS with visually impaired patients (Gordon and Felfeli, 2018). Many military veterans further compound this by avoiding disclosure of hallucinations, commonly due to fears of being misdiagnosed with psychiatric conditions such as schizophrenia, psychosis, or dementia (Dave et al., 2024; Jones et al., 2025b; Jurisic et al., 2018). When veterans do disclose, they present to a wide range of providers, including GPs, hospital doctors, ophthalmologists, and optometrists (Abdulhussein et al., 2024; Jones et al., 2025b), reinforcing the need for CBS awareness across professional settings. Within the veteran population specifically, there is an additional risk of diagnostic bias: the military background may influence diagnostic assumptions held by both healthcare providers and patients, despite CBS being unrelated to neurodegeneration (Schroeder et al., 2025).

Current clinical management of CBS centers on non-pharmacological approaches, given limited evidence for the safety and efficacy of pharmacological options—including atypical antipsychotics, anticonvulsants, cholinesterase inhibitors, and SSRIs—particularly in older patients (Hamedani and Pelak, 2019). Behavioral strategies such as blinking, moving the head, or using distraction have been anecdotally reported as helpful by some veterans, though others report little or no benefit (Christoph et al., 2024; Dave et al., 2024; Jones et al., 2025b). Education and awareness have emerged as particularly promising adjuncts: Cox and Ffytche (2014) found that prior knowledge of CBS was associated with significantly fewer negative outcomes, and Dave et al. (2024) demonstrated that understanding the neurological basis of the condition helped individuals feel more able to cope.

UK consensus guidelines recommend that healthcare professionals explain symptoms, offer reassurance, and signpost individuals to self-help strategies, and advise forewarning patients with visual impairments about the risk of CBS (O’Brien et al., 2020). However, these recommendations are inconsistently implemented in practice. A survey of 115 veterans with CBS found that just over half had received any form of management from a healthcare professional, with the most common responses being an appointment discussion (45%), an information leaflet (20%), or advice to ignore the hallucinations (9%) (Jones et al., 2025b). A retrospective audit of 155 clinical records from a UK ophthalmic outpatient clinic similarly found that only approximately 50% of patients received any CBS management, most commonly verbal advice (19%), referral to neurological or vision services (17%), or an information leaflet (11%) (Abdulhussein et al., 2024). These findings consistently indicate that, on average, only one in two veterans receives any clinical support for CBS, and that the quality and consistency of that support vary considerably. There is also limited evidence that veterans are routinely forewarned about the risk of CBS at the point of visual impairment diagnosis. Such gaps in practice may impede adjustment to CBS and contribute to heightened distress and poorer self-management (Jones et al., 2025a). According to Ffytche (p.c.), between a third and a half of people with CBS find the experience distressing and actively seek further help—a proportion that, applied to the UK veteran population, represents a substantial unmet clinical need.

### 1.4 Co-production as a tool for intervention development

Co-production is a collaborative research methodology in which researchers and key stakeholders work together as active partners throughout the research process, sharing power and expertise to develop a shared understanding of an issue and co-design effective, contextually grounded responses (Hickey et al., 2018; Vargas et al., 2022). Central to this approach is the recognition that experiential knowledge holds equivalent value to professional or academic knowledge, so interventions and resources developed through co-production are more likely to be acceptable, relevant, and sustainable for those they are intended to serve (Palmer et al., 2019; Realpe and Wallace, 2010). In veteran communities specifically, co-production approaches have improved access to alcohol misuse services, supported suicide prevention initiatives, developed veteran-led recovery groups, and assisted with identity transitions following military service (Franco et al., 2021), demonstrating both the feasibility and value of this approach with this population.

Bringing together military veterans and healthcare professionals within a co-production framework offers a distinctive methodological advantage for the present study: it enables the examination of needs, challenges, and service gaps from multiple, complementary perspectives simultaneously. NHS England (2020) specifically advocates including healthcare professionals in co-production events, recognizing that their involvement strengthens outcomes by identifying implementation barriers and ensuring that resources are realistic and deliverable within existing service contexts. A co-production approach is therefore well suited to addressing the dual challenge identified in this literature: developing resources that are meaningful and acceptable to veterans while remaining practically feasible for healthcare professionals to deliver.

### 1.5 Rationale and aims

This research was part of the Veterans’ Health Innovation Fund, funded by the Office of Veterans’ Affairs (ID 10001282), to develop a toolkit to support military veterans with sight loss who may experience CBS. It formed part of a broader programme grant, in which earlier phases quantified the prevalence of CBS among visually impaired veterans and qualitatively examined their lived experiences (Abdulhussein et al., 2024; Jones et al., 2025b; O’Brien et al., 2020). The Project Representative was Professor Renata S.M. Gomes, and the Principal Investigator was Professor Derek Farrell MBE, Northern Hub for Military Families and Veterans’ Research, Northumbria University, Newcastle upon Tyne, UK.

CBS is a common but under-recognized consequence of visual impairment in military veterans, with significant implications for emotional wellbeing and psychosocial functioning. Current clinical management is inconsistent, professional awareness remains limited, and veterans face particular barriers to disclosure and appropriate support. A co-production approach—bringing together veterans with lived experience and healthcare professionals with service knowledge—offers the most contextually responsive means of developing resources that address this gap from both perspectives. The aims of the study were therefore as follows:

1. To explore experiences of managing CBS and accessing healthcare services and provisions, from the perspective of military veterans living with CBS and healthcare professionals.

2. To create a toolkit to support military veterans and healthcare professionals in managing CBS and accessing healthcare services and provisions.

3. To gather stakeholder perspectives and feedback on the initial toolkit developed and to implement recommended alterations.

4. To explore the possibility of an intervention piece for military veterans with CBS.

## 2 Materials and methods

The “consolidated criteria for reporting qualitative research utilizing interviews and focus groups” (COREQ) (Tong et al., 2007) checklist was used to guide the reporting of this paper. The study was reviewed and approved by the Northumbria University Ethics Committee (Ref: 9370).

### 2.1 Approach

A co-production approach was used to bring key stakeholders (military veterans and healthcare professionals) together as active and equal partners to contribute to a shared understanding of the topic and to co-produce and refine a toolkit. To achieve this, two workshops were conducted. The first co-production event, Phase 1, involved conducting a series of qualitative focus groups situated within a structured co-production workshop. The aim of Phase 1 was to understand the range of experiences and perspectives on managing CBS, accessing healthcare services and provisions, and to identify areas of need. Based on feedback from Phase 1, an initial toolkit was developed and then presented for feedback during the co-production Phase 2 workshop. In Phase 2 of the co-production, another qualitative focus group was conducted to gather opinions and perceptions on the toolkit and identify areas for improvement.

### 2.2 Research team and reflexivity

This qualitative study is grounded in a constructivist paradigm, which acknowledges a collaborative construction of knowledge between participants and researchers (Denzin and Lincoln, 2011). The co-production event was facilitated by MK, who has extensive experience in undertaking co-production events. DF, RSMG, ES, JA, and JM conducted focus groups. JM and DF led the analysis; all authors have prior experience in qualitative research. We recognize that the professional and personal experiences held by each member of the research team have the potential to both enrich and bias the research process. To address this, we maintained a reflexive stance (Hibbert et al., 2014) throughout the study, regularly discussing our perspectives and decisions within the team to ensure a balanced and thorough analysis.

### 2.3 Participants

Purposive sampling was used to recruit both military veterans and healthcare professionals, consistent with co-production methodology, which prioritizes meaningful representation of relevant stakeholder perspectives rather than population-level saturation (Hickey et al., 2018; Patton, 2014). All participants were required to be over 18 years of age and either have a confirmed diagnosis of CBS or work professionally with individuals with CBS.

Military veterans were recruited through Sight Scotland Veterans (SSV), a charity supporting visually impaired veterans in Scotland. Visual impairment-friendly recruitment documents were provided and discussed verbally by SSV support workers with veterans attending an SSV activity hub; consenting veterans were supported by SSV with transport to and from the workshop venue. A target of 10 veteran participants was set, reflecting the clinically specific and hard-to-reach nature of this population—military veterans with confirmed CBS and visual impairment—and this target was met. Healthcare professionals were recruited through the personal and professional networks of the research team and SSV, with invitations extended to those working in visual impairment and veteran health contexts. Thirteen specialists were recruited, exceeding the initial target of 10 and ensuring representation across a broad range of professional roles, including vision rehabilitation specialists, occupational therapists, general practitioners, medical welfare officers, support workers, and helpline support workers. No remuneration was provided to participants at any phase of the study.

Consistent with the iterative character of co-production research, analytical attention was paid throughout both workshops to whether new themes or perspectives were emerging. No substantively new issues arose in the later stages of either workshop, providing assurance that the depth of stakeholder coverage was sufficient for toolkit development.

Of the 23 recruited participants, 7 military veterans and 13 healthcare professionals participated in Phase 1, and 4 military veterans and 5 healthcare professionals participated in Phase 2. Reduced veteran attendance at Phase 2 was attributable to non-attendance at the SSV activity hub on the day of the workshop; additional veterans were therefore recruited to provide feedback on the initial toolkit at that stage. Individual demographic data for military veterans were not collected.

### 2.4 Co-production research methodology

This study employed a co-production research methodology, guided by the NIHR framework for patient and public involvement and the principles articulated by Hickey et al. (2018), which position co-production as a collaborative process in which researchers and stakeholders share power, contribute complementary expertise, and jointly shape both the research process and its outputs. As Sklar et al. (2025) exemplifies, co-production is motivated to produce a mutually beneficial outcome that is jointly developed by two or more actors. Consistent with these principles, co-production was understood not as a consultative add-on but as a foundational epistemological commitment. Military veterans and healthcare professionals were engaged as active and equal partners throughout the study, contributing lived experience and professional knowledge as forms of expertise of equivalent value to academic knowledge (Slay and Stephens, 2013). This approach was selected because the research question, concerning the real-world experience of managing Charles Bonnet Syndrome (CBS), and the design of accessible support resources, is inherently practice-embedded and could not be adequately addressed without the sustained involvement of those who navigate these experiences directly.

**FIGURE 1:**
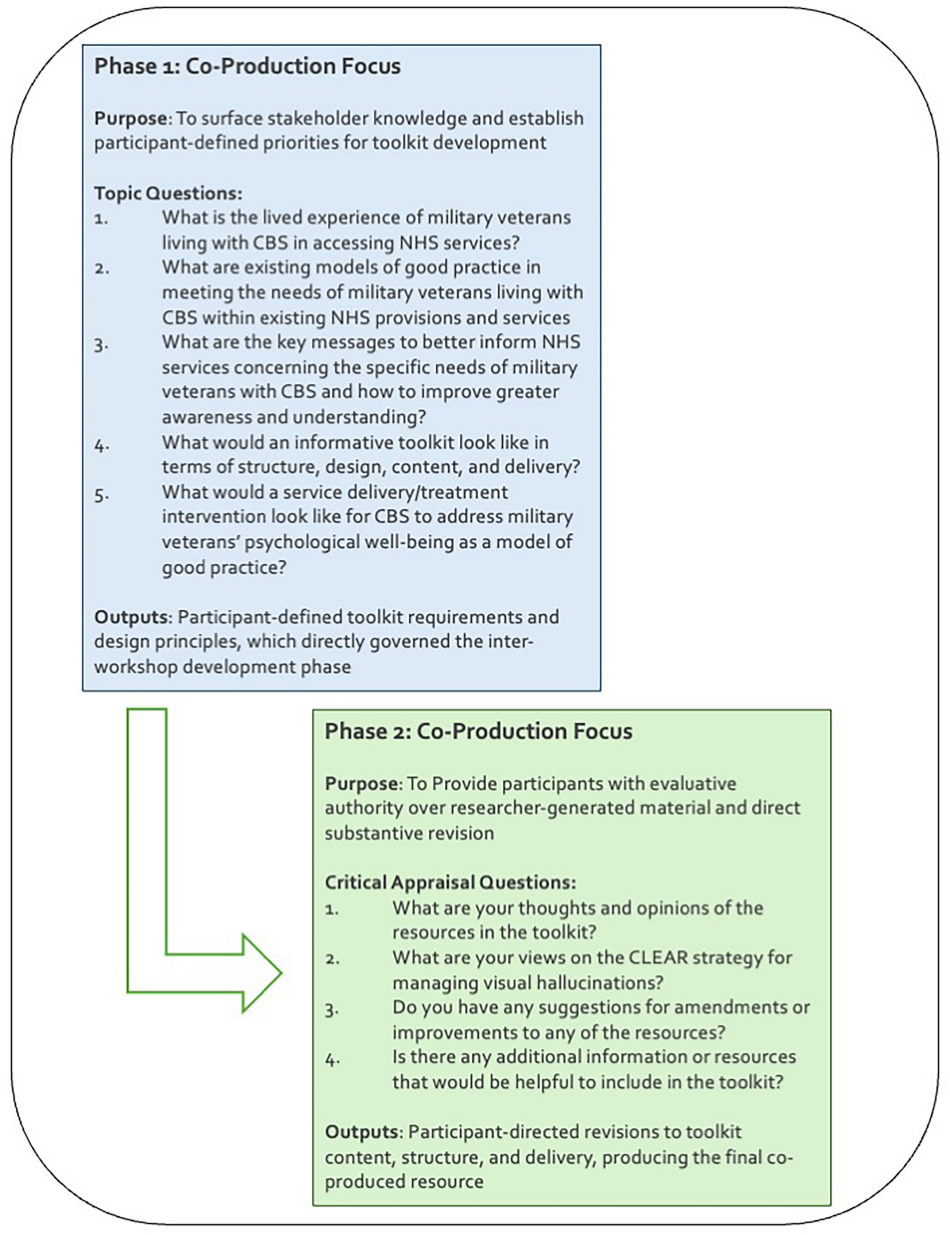
Co-production question frameworks used across Phase 1 and Phase 2 workshops.

To operationalize these principles, two structured co-production workshops were held, each iterative and bi-directional. Findings and participants’ contributions from the first event directly informed the research team’s subsequent actions. Furthermore, the outputs of those actions were returned to participants for critical appraisal, refinement, and approval at the second workshop. This design reflects what Greenhalgh et al. (2019) describe as “adaptive” co-production, in which the research process remains genuinely responsive to participant input rather than following a pre-determined researcher-led trajectory. The core elements of each phase is outlined in Figure 1.

#### 2.4.1 Phase 1 co-production workshop

The first co-production workshop (Phase 1) was held at a Support for Sight Veterans (SSV) activity hub in Scotland, UK. The venue was selected in consultation with veteran participants to ensure accessibility and familiarity with the setting. The workshop began with introductions from the research team, followed by a brief overview of previous research on CBS in military veterans conducted by colleagues in the broader research team. The research project’s aims and the workshop’s structure were outlined, and participants’ questions were addressed before proceedings commenced.

Participants were then divided into three groups, each deliberately balanced in composition across military veterans, healthcare professionals, and researchers. This compositional parity was a structural mechanism to prevent researcher dominance and ensure that lived experience and professional knowledge held equivalent standing in group deliberations, consistent with the asset-based principles of co-production (Realpe and Wallace, 2010). Each group was facilitated by MK, who adopted a process-facilitation role, guiding discussions and prompting elaboration rather than a directive or expert role, explicitly positioning participants’ knowledge as primary.

A semi-structured question guide was developed to facilitate workshop discussions (Figure 1), and accessible copies were provided to participants in advance of the workshop. Distributing questions in advance was a deliberate decision to equalize participation: it gave veterans, some of whom experience visual impairment, and those less accustomed to academic workshop formats, the opportunity to prepare contributions on their own terms, reducing barriers to equitable engagement. During the workshop, questions were displayed on screens, and printed copies, including large-print versions, were available at each table, following individual preferences and established guidelines for producing materials for people with sight loss.

The first topic question, “What is the lived experience of military veterans living with CBS in accessing NHS services?,” was introduced, and each group discussed this for 30 min. The second topic question, “What are existing models of good practice in meeting the needs of military veterans living with CBS within existing NHS provisions and services?,” was then discussed for a further 30 min. Following these group discussions, the groups reconvened and the research leads for each group (DF, ES, JA) presented summaries of the key points raised. Critically, these summaries were not treated as researcher-authored findings but as a shared resource for collective sense-making: participants were invited to affirm, challenge, or extend them, ensuring that interpretive authority remained with the group rather than the researchers.

After a lunch break, the groups reassembled to consider the third topic question: “What are the key messages to better inform NHS services concerning the specific needs of military veterans living with CBS and how to improve awareness and understanding?” Following 30 min of discussion, the fourth topic question, “What would an informative toolkit look like in terms of structure, design, content, and delivery?,” was introduced and given 45 min for discussion. The fifth and final question, “What would a service delivery/treatment intervention look like for CBS to address military veterans’ psychological wellbeing as a model of good practice?,” was discussed for 30 min. Following each set of group discussions, research leads again reported back to the whole group, with summaries used as a basis for collective deliberation rather than final conclusions.

To conclude the workshop, each participant and member of the research team was invited to share one key take-home message, summarizing what they felt was most significant about the workshop. This closing exercise served as both a reflective summary and an equity mechanism, ensuring that each voice, regardless of professional role or academic background, held equal standing in articulating the session’s significance. Each focus group discussion was audio-recorded using a Dictaphone, and the full-group feedback and discussions were similarly recorded. Researchers also took detailed field notes during the focus groups. All recordings and field notes were collected for later transcription and thematic analysis. Following the participants’ departure, the research team held a structured debrief meeting to reflect on the workshop process, identify areas where participant contributions had redirected or refined the emerging research agenda, and agree on a development plan for the toolkit.

#### 2.4.2 Inter-workshop development phase

In the weeks following Phase 1, the research team undertook the analytical and developmental work of producing an initial toolkit draft. This phase necessarily involved researcher-led activity, as the team conducted thematic analysis of workshop recordings and field notes and translated participant-generated priorities into draft resources. This is acknowledged as a stage of the co-production process in which participant involvement was less direct, and we treat this as a transparency obligation rather than a methodological weakness: hybrid co-production models, in which intensive collaborative phases are interspersed with researcher-led analytical work, are well documented in the literature and considered appropriate where participant time and capacity are limited (Staley, 2009; Greenhalgh et al., 2019). Critically, the thematic outputs and design decisions made during this period were determined by participant contributions from Phase 1 rather than by researcher preference, and all outputs were subsequently returned to participants for scrutiny and revision in Phase 2.

#### 2.4.3 Phase 2 co-production workshop

Once the draft toolkit was complete, all Phase 1 participants were contacted by email or via an SSV support worker, depending on their preference, and invited to attend a second workshop (Phase 2) to review and provide feedback on the draft. The use of multiple contact channels was intentional, reflecting a commitment to co-production in reducing participation barriers and ensuring access was not restricted by digital literacy or communication preferences. The Phase 2 workshop took place six weeks after Phase 1, again at the SSV activity hub.

The workshop began with a recap of the key toolkit requirements and design features identified during Phase 1, explicitly reconnecting the group with their prior contributions and signaling that the draft was accountable to those contributions. The research team then summarized actions taken since the previous meeting and outlined the contents of the draft toolkit. Toolkit resources were presented one by one, and a structured group discussion followed each resource. These discussions were explicitly framed as evaluative and revisionary rather than confirmatory: participants were positioned as critical assessors with authority to identify inadequacies and direct changes. Discussions focused on the relevance, clarity, and usability of each resource, and participants were encouraged to provide detailed suggestions for refinement.

Again, the entire workshop discussion was audio recorded, and researchers took field notes throughout. Any written comments provided by participants were collected at the end of the session. The workshop lasted approximately three hours. Recognizing that some Phase 1 participants were unable to attend Phase 2 in person, healthcare professionals who had participated in Phase 1 but not Phase 2 were subsequently contacted by email and provided with copies of the draft resources, with an explicit invitation to submit written feedback. All correspondence received was incorporated into the dataset and subjected to the same analytical process as workshop material, ensuring that the perspectives of those unable to attend were not lost.

### 2.5 Reflexive considerations

We acknowledge that co-production exists on a continuum (Arnstein, 1969; Greenhalgh et al., 2019), and we do not claim that every element of this study achieved an idealized model of equal co-authorship of research decisions. As Sklar et al. (2025) highlights, co-production as a research methodology creates multi-level challenges and opportunities in creating integrated care implementation. Researchers designed the initial question guide, led thematic analysis, and produced the first draft toolkit, roles that carried inherent authority. However, we contend that the structural mechanisms employed across both workshops balanced group composition, facilitated advanced question sharing, enabled participant-led cross-group synthesis, allowed iterative return of outputs for participant scrutiny, and enabled inclusive multi-channel participation. Collectively, these mechanisms constituted a robust and substantive co-production process in which participant expertise shaped both the direction and the outputs of the research in ways that would not have been achievable through conventional researcher-led design. We present this account with transparency rather than claiming a more idealized model than the constraints of time, access, and participant capacity permitted.

### 2.6 Transcription process

Data from Phase 1 and Phase 2 were transcribed verbatim by Otter.ai. (2025), a transcription service that uses artificial intelligence to convert speech to text. Each transcript was then manually checked and edited against the audio file for accuracy (JM). Any identifying information in the transcripts was anonymized before being uploaded to the qualitative software tool NVivo (version 14) (Lumivero, 2023) for analysis.

### 2.7 Data analysis

Transcripts and field notes were analyzed using inductive reflexive thematic analysis (Braun and Clarke, 2006, 2021). A separate analysis was conducted for each phase of the study. For both phases, following familiarization with the dataset, semantic coding commenced on NVivo. Coding was completed in full by two researchers (JM, DF), with codes reviewed and refined based on team discussions (JM, DF, RSMG). Initial patterns considered pertinent to the research aim were compiled into preliminary themes (JM, DF), which were further reviewed and revised with the broader research team (JM, DF, RSMG, MK). Agreement on the names and definitions of final themes was reached through discussion amongst the research team. The rigor of the analysis was enhanced through repeated readings of transcripts by multiple team members, individual and collective reflexivity throughout the research process, and regular team discussions to refine emerging findings.

## 3 Results

### 3.1 Phase 1 findings

The primary objective of Phase 1 was to explore the experiences of living with and managing CBS, as well as accessing healthcare services and provisions, from the perspectives of military veterans and healthcare professionals. As part of this, three themes were identified. The first theme, “The reality of living with CBS,” highlights military veterans’ personal experiences of visual hallucinations, the variations in which individuals emotionally and psychologically respond to these visions, and the personal and societal stigma that often surrounds the experience. The second theme, “Management of CBS,” outlines the strategies employed by military veterans and healthcare professionals to manage visual hallucinations, along with the challenges they encounter and the areas where additional support and resources are needed. The third theme, “Barriers to accessing healthcare,” highlights the lack of awareness of CBS amongst general healthcare professionals, the gaps in ophthalmology services, and the important role that sight loss charities play in bridging these gaps in support. Table 1 highlights quotations to support the themes of living with CBS. Table 2 outlines quotations in support of the themes to develop the Toolkit.

**TABLE 1:** Quotes to support the themes of ‘The reality of living with CBS’, ‘Management of CBS’ and ‘Barriers to accessing healthcare’

The reality of living with CBS	Experiences of visual hallucinations	“*I saw mice running across*[the floor]*, had they of been real I wouldn’t have seen them, I’d have been out the door, but I knew they weren’t, ‘cos all of a sudden they would disappear… I knew that they would disappear, they weren’t going to follow me or anything*” – MV
		“*When I go to bed, I leave the table light on beside the bed, all night, because I find if it’s dark it could happen, if the lights are on, it never seems to happen*” – MV
		“*I mean I, I suffer virtually, not every day, but most days with hallucinations in some shape or form. I mean the ones when you’re eating something, corn flakes and you see something*” – MV
		“*When I’m watching the television, I can see people on the screen, on the television programme… I can see wee men and women walking along the screen in the actual television picture*” – MV
		“*If I’m sitting beside somebody that’s driving and you see somebody crossing, you know it could be two women with children crossing roads, and they walk right in front of you, and you tell the driver and they’ll say there’s no one there*” – MV
	Hidden impact of visual hallucinations	“*Some people enjoy them*[visual hallucinations]*. I had a woman who saw baskets of flowers and she quite enjoyed it. Another woman saw a German shepherd dog in the middle of her living room, and she said, ‘It doesn’t bother me, I just get my brush and I just brush him away.’ So it varies, another chap, it was a tiger at the window, which did terrify him, you know that, that would terrify him*” – HCP
		“*I was trying to get a man to go to the shops and it was just across the road, but cars would come down quite quickly, so we both went further down the road to try and get across, and then he seen a bicycle in the middle of the road, so he was waiting and he just kept waiting and waiting, and so it is a physical disability to get across the road, that’s how real it is, you know*” – HCP
		“*One veteran was absolutely terrified, he had to move his flat, his home and all the rest of it, because he was totally seeing things and he thought they were real, and he had to go into a home, that way he knew that the people round about him were real*” – MV
		“*I was scared at the beginning because I didn’t know what it was, and I really thought, ‘Oh, that’s great, as well as going blind I’m now starting to lose my mind’, you know, and you didn’t feel able enough to disclose about the rabbits, and that, that was part of your own fear and anxiety about this, this maybe is dementia*” – MV
	Stigma surrounding visual hallucinations	“*I kept seeing the little rabbit that kept appearing in my living room and I kept it a secret for six months. I didn’t, didn’t know that it was, I knew it wasn’t real, and I thought, if I’m going demented, I’d think it was real and try and feed it. So I know it’s not real, but why do I keep seeing it? And it was finally, when I was at my consultant, she said, ‘Do you have any other problems?’ So I thought, she probably doesn’t have a strait jacket here, so I’ll risk it*” – MV
		“*So if you think 85, and you hear the words, ‘seeing things’, most family members are going to think Mum or Dad have a cognitive decline*” – HCP
		“*They don’t want to admit they’re seeing things because they’re expecting the men in white coats to come and start taking them away*” – HCP
		“*If somebody came to me, and it was one of my clients, and they were saying, ‘I’m also seeing things’, I’d have referred it straight into mental health*” – HCP
Management of CBS	Practical strategies used to manage CBS	“*When I feel tired, I just go and sit down and then maybe when I open my eyes, that’s when I see these things. I try and stop doing things before I get tired now, I mean, I just started going to my bed and lying down just for a couple of hours, and I never would have done that*”– MV
		“*If you blink your eyes, I’ve been lucky, if you blink your eyes the next thing you know… they’re gone*”– MV
		“*Music can calm you down, depends on what your taste in music is, and as I was saying, boom radio, that goes way back over the years with disc jockeys that have retired and they play music, and you can say ah I remember, and I was doing such and such a thing*”– MV
		“*So we*[SSV] *did a 10-minute relaxation USB stick for folk as well, and it seemed to help them with it*[visual hallucinations]”– HCP
		“*When we’re in the transport, he’ll say, ‘do you see that big building over there?’ and I’ll say ‘no, I don’t see it’, and then I’ll say ‘do you see that over there?’, and he’ll say ‘no, not really’, and we’ll laugh. We very rarely, if ever, see the same thing*”– MV
		“*I think the fact that erm, that you know, that somebody says, ‘have you ever experienced this?’, and I’d say ‘iye’, then I would say to them ‘well, do you want me to find someone that can explain it to you?’, you know try to help the person*”– MV
	Challenges in managing CBS	“*You shake your head and everything, you try to get it away, and I put a wee, I do my eyelashes to see if that goes away, sometimes it works, sometimes it doesn’t*”– MV
		“*One lady that I’ve been working with quite recently suffered full and sudden sight loss and she has started to experience Charles Bonnet, but because of the full sight loss she doesn’t have the ability to use the distraction techniques of maybe switching a light on and off, or, you know, the TV on and off, so those visual distractions she can’t use*” – HCP
		“*I’ve got veterans that, generally will see things and know that they don’t exist, right, has a train that goes through his bedroom every morning, until he’s at a train station and that train comes along and he reacts to a person that’s close to a railway, but he’s obviously really, really aware as well, that’s he’s going to potentially look mad, because the context is, in a bedroom, I’m not going to get a train going through the middle of my bedroom. I’m at a train station, and I’m getting that context, how do I react to it?*”– HCP
		“*See as a practitioner, when we can’t find something that helps, I find that really, really difficult, because you still, you’ve just told the person what potentially is going on, you’ve told them all these coping strategies that other people are finding useful, and then it doesn’t work for them, and you go ‘Okay, cheerio now’*”– HCP
Barriers to accessing healthcare	Perceived lack of awareness of CBS amongst general healthcare professionals	“*So if that came through to screening for me, so if it just had, whatever, the difficulties were, maybe hallucinations, elderly, I would put that in an older persons team, if it came through and it had the same, outcome of this is distressed, complex, difficulties at home, I would put that under mental health*”– HCP
		“*Because I work with dementia, you know, if I had, you know, came to see you, I would query if, if you had a memory problem or hallucinations in connection with dementia*”– HCP
		“*MV: I had to get a new GP, because I phoned in one morning and he said, ‘I’m just looking at your chart, you’ve seen four different doctors and they’ve given you four different diagnoses, from now on, I’m your main doctor’. R: And how did you feel after getting four different explanations? MV: Well, you got to the stage where you were confused*” – MV & R
		“*I was supporting this man once, he was in A&E at the time, he was really, really quite bad with other stuff, but erm, the doctor had no clue, I had to sit and explain to the doctor what Charles Bonnet was*” – HCP
	Gaps in ophthalmology services	“*The doctor at the eye clinic told me about it*[eye condition/ visual impairment]*, but he never mentioned anything about any side effects that you might see things, he just said this will obviously detract from your direct vision…but there was never any talk about seeing anything at all actually…they were more concerned with saying the things you’ll lose, you’ll not drive, obviously, I can’t read anything or computers or anything electronic*” – MV
		“*Generally, what will happen is, when there’s nothing else that can be done, that’s you registered, you don’t need to come back, so, maybe CBS starts up after that, so who’s supporting people after that? What they should do is go to their local optician, but not, how many people do we see that don’t go for regular eye checks?*”– HCP
		“*Nothing can treat it once it’s happened*[visual impairment]*, so he’s just been discharged, and there’s nobody then after that, so, until the likes of us*[SSV] *would come along, but he could wait long enough for a rehabilitation officer to come along in his life to explain what that could be, and in that time, he could be absolutely frightened to death*”– HCP
		“*So people can be waiting a long time before they get to an eye clinic, before they get this information. When they do get to the eye clinic, the ECLOs, so these are the eye clinic liaison officers, but not all of them*[eye clinics]*have them. I mean, they should do it, it is an NHS standard, but they don’t, and then they’re not always there, and some aren’t even aware of it*[CBS]” – HCP
		“*I used to work in an eye clinic, it’s such a busy, busy place, I never thought about how that person got to the clinic, I just measured their vision at the end of the day, put some drops in and what not, but it was just so busy, you were in and out, in and out, in and out, you never thought about how they got there or what they were experiencing*” – HCP
	Sight loss charities bridging the gap	HCP1: “*Can I tell you who normally diagnoses? Us. It’ll be us*[SSV rehabilitation officers]”. R: “*But you’re doing that as a rehab officer?*” HCP: “*We’re doing that as rehab officers*…”. HCP2: “*We have too because*…”. HCP1: “…‘*cos nobody else is asking*”– HCPs & R.
		“*We’re not medical professionals, we’re rehab officers, but when visiting veterans at home and we ask the question, ‘are you seeing things you know aren’t there?’, they’ll say ‘Yeah. I’ve been frightened to say anything to my family.*” – HCP
		“*I think the important thing is that people have, you know, get the appropriate support, and we make sure it’s a proper diagnosis, so people really do need to see their GP, so we’re not presuming it’s Charles Bonnet Syndrome, but that it could be a medical problem as well, so we always recommend ‘look, it could be, but go see your GP’* “ – HCP
		“*We ask the question, ‘Do you see things that you know are not there?’ Every time we do an assessment with somebody, with a veteran at home, we’ll say, ‘Okay, when did this start? Have you had a change in your vision lately? Are you on any new medication?’ ‘No’ ‘No new medication, okay’, so we can kind of scrap that as a cause*” – HCP
		“*We’ve*[SSV]*also done, occasionally, we do talks, we come into the centre, and we, we get together with some veterans, and they ask them if they want to have a talk about CBS. I’ve been to community groups, and have spoken to the vision support groups that are run by our independent living workers, and we chat about it there as well*” – HCP

CBS, Charles Bonnet Syndrome; HCP, Healthcare Professional; HCPs, Healthcare Professionals; MV, Military Veterans; R, Researcher.

**TABLE 2:** Quotes to support the themes of ‘Development of a toolkit and ‘Refinement of a toolkit’.

Development of a toolkit	The focus of the toolkit	“*So I guess from a, from a professional point then, the key areas are probably around awareness raising, whether the presentation is this or this, and actually, are we asking those questions at presentation, and erm, staff having the knowledge, and I mean, I suppose it’s not just A&E staff, really, it can be, it should really be any, any…any healthcare staff really*” – HCP
		“*That*[informational resource]*would actually be beneficial, because people who have it, they don’t want to talk about it, because people they’re talking to don’t understand what they’re talking about in the first place, so it’d be nice to have communication from somebody who knows about it*” – MV
		“*I really think there should be something out for families to understand that what the member of the family is experiencing, isn’t necessarily to do with dementia or anything else, I think that’s important*” – MV
		“*They’re*[healthcare professionals]*not necessarily ruling it out because they’re maybe not aware of it, that’s the thing, so it’s like that baseline knowledge of just asking the question around vision loss that would then trigger about CBS for somebody with hallucinations*” – HCP
		“*There should not just be one*[toolkit]*, because I think the information that would be acquired, depending on who you are in the pathway, would be, it would be different information that should be in there, and it would be more useful if it was kind of tailored to them, whereas if you were to just take the same thing that you would randomly give to a consultant, I don’t know if that would be accessible to a person that’s not a doctor. So in my mind, I don’t know how effective it would be to have one single toolkit for every single group*” – HCP
		“*So we could have something that would be tailored for the veteran to have, a memory stick that they can kind of put in when, if, they were to feel distressed, almost like a safety plan that you would do in a time of crisis, but for this individual, when I feel distressed, these are the things I would do, these are the things that work for me and that, I guess you would have learned from what’s worked from others, from exploring together, from the support groups, I would imagine a lot of the topics look at coping. I would imagine that would be the best approach if we were thinking about an intervention*” – HCP
	Required features of the toolkit	“*It’s got to be audio, I mean, a leaflet is totally useless, because I got a dozen leaflets in a week, advertising everything, and they all went to the bin, and it’s because I can’t read them. I can tell colours, but I can’t form figures on letters*” – MV
		“*If you get an audio memory stick, or audio book, some of the voices bore you to tears, you would give up and probably stop using it because of the voice, whilst you’ve got others and er, they actually, where they actually accentuate the words and act the part, in fact, act the part …the voice needs to be engaging, because if it’s not, you tend to lose interest*” – MV
		“*So my problem is I can lose a point quite quickly, so I think if you can build on something, and it stops, and then recap it, then go into the next bit, just snippets and then have the podcast over four or five different snippets with bite-sized pieces of information*” – MV
		“*I always remember stats, like one in two people have cancer, I remember that, so one in five, you know, just to normalise it. I also think it should include the lived experience of some stories, good and bad, scary and normal stories, beautiful ones will also help normalise the experiences*” – HCP
		“*It’s important to say, though, if you just go down the audio route, you’re excluding people that cannot hear very well, so you need to have, so there would need to be leaflets as well, in an accessible format*” – HCP
		“*You know what would be really good in the leaflets, to have visual pictures so, really, for people to kind of try and understand, and they talk about like gargoyles and kids in Victorian dress being quite a common hallucination*” – HCP
Refinement of a toolkit	Initial perceptions of toolkit	“*It would have been good to have had at the beginning because then you would have known straight away, oh, this is a natural thing that is happening, rather than, you know, bearing in mind that I’m in such a state of shock at what has happened to me, to then think, within a couple of days, not long ago have I lost my sight, I’m now starting to lose my mind*” – MV
		“*I think it’s very good, very informative. I wish I had heard it when it first happened to me, because the way I lost my sight, literally overnight, my body didn’t adjust easily*” – MV
		“*I think it was just about the right length, because with the jingle in between, you know you’re going on to a different section, so it just gives you those few seconds to assimilate what you’ve heard before. And I liked the clear thing, it helps you know what to do when you have one, but I’m glad they went over it again at the end, because it clicks a bit better, because that was that worked for you the first time you hear it, you think, no, I’m never going to remember that, you know, so when it’s repeated at the end, that’s good*” – MV
		“*The clear acronym, I just think that’s wonderful. I’ve got a big tick next to that. It’s one that will stick in our head and the veterans, it’s vision related, it’s quite easy to just kind of sit and go through with someone, especially when you’re trying to deliver that to a person that is non-medical, but also like, you know, the people that we’re working with*[other healthcare professionals]*. So if you’ve got something that you can just go to, you know, it’s like the fast one, isn’t it, that stroke one and you remember it because of that, so I think that’s brilliant*” – HCP
		“*I think the one for healthcare staff is good as it goes through it*[CBS] *in depth, and it covers a lot of the things they need to know to actually be able to help people, you know, like knowing how to spot it and then how to actually manage it, which can only be a good thing*” – HCP
	Suggestions for improvements	“*So what they* [College of Optometry] *now do, is they do short videos of conditions that you can actually share, again, it’s making it more accessible, so rather than giving out a patient leaflet, or, in conjunction with that, you can actually send people a short video, doing something like that would be brilliant*” – MV
		“*Just thinking, if you put on like, if you link on to the likes of Instagram, TikTok, and you put on through the NHS inform, or whatever, you could link your, your podcast onto that, so, you know, it’s a, normally only what 30 seconds, aren’t they, they’re little short videos, but you could link that on, like, because I’m just thinking of the way that I listen to podcasts, you’ve seen clips, and then I’ll link onto them to go and listen to them. So that would be excellent*” – HCP
		“*The vast majority probably just need information, the right information, in the right way, in the right format, but there’s another group of people, that actually they’ll need more than that, they’ll need something to help improve their ability to regulate, particularly, if they find it sort of distressing, like severe extreme palpitations, and like a severe trauma response, that will need something more like psychological trained support*” – HCP
		MV: “*I’d like a more condensed version, it’s still a lot of information to try and absorb, and it’s like anything you get to a certain age, you can’t, it doesn’t stick.*”R: “*Just to be clear what you’re saying then, is that what might be useful is an even shorter version?*”MV: “*Yes, I think, I like the main aspects of it, I just think short and sweet, like three, four minutes*” – MW & R
		“*So an idea then coming from that, that there’s a leaflet produced that we can leave with the veterans during visits, who can then go to their GP with it*” – HCP

CBS, Charles Bonnet Syndrome; HCP, Healthcare Professional; HCPs, Healthcare Professionals; MV, Military Veterans; R, Researcher.

#### 3.1.1 The reality of living with CBS

##### 3.1.1.1 Experiences of visual hallucinations

Military veterans with CBS reported experiencing a wide range of complex and static visual hallucinations. The static visions tended to be of simple shapes or lights, while others experienced a continual overlay, such as a brick wall, that occurred opaquely across their vision. The more complex visions tended to move and typically involved images such as figures (e.g., gargoyles or figures dressed in Victorian clothing), animals (e.g., dogs, butterflies, rabbits, insects), flowers, or plants. Some visions aligned with the contextual setting, for instance, a vase of flowers in the kitchen, a train appearing at a train station, or a person/bicycle crossing the road. Other visions were more intrusive, such as figures appearing at the bottom of the TV screen while watching a programme, or insects crawling on food or across the floor. There was variation reported in the frequency of a visual hallucination; for some, they occurred daily, while others experienced intermittent episodes over several years. A few veterans noted that hallucinations were more likely to occur in the dark, prompting them to sleep with the light on. Despite the vivid nature of the hallucinations, most veterans were able to distinguish them from reality, often recognizing through logic or context that what they were seeing was not real. However, this was a much more challenging task when the moving vision matched the environmental setting (e.g., train at the train station).

##### 3.1.1.2 Hidden impact of visual hallucinations

There was variation in the emotional and psychological responses reported by the military veterans and the impact that visual hallucinations had on their daily lives. For instance, many of the veterans reported feeling unbothered or unalarmed by the hallucinations and displayed a stoic attitude of just getting on with it. However, vision rehabilitation officers noted that many other veterans within community settings did find the hallucinations highly distressing. It was reported that the emotional impacts often depended on the nature of the hallucination itself. For instance, seeing something benign, such as a butterfly or flowers, tended to be less distressing than encountering more alarming images, such as a tiger or a train coming through the bedroom. In some cases, the hallucinations had significant practical consequences, such as contributing to a move from independent to supported living due to the emotional distress and psychological impact directly because of the hallucinations being experienced. An additional source of distress occurred when individuals first began experiencing visual hallucinations, particularly if they were unaware of CBS. This lack of awareness often led to fear and anxiety, with many concerned that the hallucinations might be a sign of cognitive decline. This unknowing led to catastrophizing behaviors which intensified emotional and psychological distress.

##### 3.1.1.3 Stigma surrounding visual hallucinations

Many military veterans experienced a strong sense of stigma attached to experiencing visual hallucinations. They were very aware that their experiences could be mistaken for signs of dementia or cognitive decline. This caused a reluctance to disclose their experiences with family members and healthcare professionals because of the negative connotations attributed to visual hallucinations. In the instances where military veterans have disclosed their symptoms to family members or healthcare professionals, they tended to add disclaimers to the conversation (e.g., “that they are not going mad, or that it is not dementia”) as a way to be taken seriously. However, these strategies were not always practical, as some family members still developed negative preconceptions about the individual. In some cases, healthcare professionals unfamiliar with CBS referred veterans to mental health or dementia services. These responses not only reinforced feelings of stigma but also led to additional emotional distress.

In summary, military veterans with CBS reported a wide range of visual hallucinations, which varied in content, frequency and emotional impact. While some veterans adopted a stoic attitude, others, particularly those without prior knowledge of CBS or who were not as well integrated into sight loss charities and support services, experienced higher levels of emotional and psychological distress in response to visual hallucinations. The lack of knowledge and awareness of CBS led some individuals to avoid disclosing their experiences to family and healthcare professionals, fearing negative judgment and misdiagnosis. These findings underscore the importance of raising awareness of CBS among military veterans, their families, healthcare professionals, and the broader communities in which individuals with CBS reside. This could help reduce stigma, alleviate distress, raise awareness and encourage the disclosure of symptoms and experiences.

#### 3.1.2 Management of CBS

##### 3.1.2.1 Practical strategies used to manage CBS

Military veterans shared a variety of personal strategies they use to manage visual hallucinations. Many individuals described using behavioral techniques such as blinking, moving their head or eyes, swiping at the vision with their hand, or changing their environment (e.g., moving to another room, adjusting lighting, or engaging in a different activity) to help interrupt or alleviate visual hallucinations. Listening to their favorite music was also commonly used as a distraction technique to help redirect their attention from the vision. Military veterans also discussed being aware of their personal triggers, for instance, noticing that they were likely to experience hallucinations when they were tired, fatigued or stressed. This awareness helped them take proactive steps, such as pacing their daily activities, taking regular naps, or engaging in activities that destressed them (e.g., watching their favorite TV programme), to reduce the likelihood of a hallucination occurring. Rehabilitation officers also reported that relaxation techniques and mindful breathing techniques were helpful strategies that they advised military veterans to engage with to help relieve hallucinations. Although not a practical strategy, veterans also highlighted the value of peer support, noting that having conversations with others who were also experiencing CBS helped them feel less alone. It was also an opportunity for them to exchange tips, share resources, and contact details for avenues of support. In some cases, military veterans used each other to help determine whether a vision was real, often employing humor to lighten and normalize the situation, thereby easing emotional discomfort.

##### 3.1.2.2 Challenges in managing CBS

While many military veterans reported using behavioral techniques and distraction strategies, many noted that there was variability in the effectiveness of these strategies, whereby sometimes they worked to clear the hallucination and sometimes they did not. For others, these strategies were consistently ineffective. Both military veterans and healthcare professionals emphasized the need to have alternative strategies available in instances where the usual strategies were ineffective, as well as strategies for when the visual hallucination matched the environmental context, particularly when the ability to distinguish the vision as a hallucination was low. There was also a recognition of the need for tailored approaches for individuals with minimal vision or complete blindness, for whom behavioral techniques such as blinking, eye movements, or adjusting lighting had no effect. Healthcare professionals also highlighted a lack of strategies to help military veterans manage the psychological and emotional impacts of CBS and visual impairment, particularly in supporting adjustment to both conditions.

In summary, military veterans and healthcare professionals described a range of strategies to manage visual hallucinations, including behavioral techniques, distraction methods, relaxation exercises, and peer support. However, several challenges were identified, particularly around the inconsistent effectiveness of these strategies, which varied between individuals and across different situations. There was also a lack of strategies available to help military veterans adjust to both sight loss and CBS, as well as for managing the associated psychological and emotional distress. Overall, these findings suggest that a “one-size-fits-all” approach is insufficient and that resources should offer a variety of strategies to account for such variations while also supporting emotional wellbeing and adjustment needs.

#### 3.1.3 Barriers to accessing healthcare

##### 3.1.3.1 Perceived lack of awareness of CBS amongst general healthcare professionals

Participants commonly perceived low awareness of CBS amongst general healthcare providers, particularly within GP settings. This lack of awareness was associated with difficulties in receiving accurate information, delays in diagnosis, and inappropriate referrals to other services. For instance, without knowledge of CBS, the healthcare professionals often misattributed hallucinations to other conditions such as dementia, post-traumatic stress disorder (PTSD) or mental health issues. There was a tendency for GPs to make referrals based on other presenting factors. For instance, if an individual were struggling with self-care, they would be referred to older persons’ care teams. Similarly, if an individual were noted as being stressed, they would be referred to mental health teams. As a result, many military veterans reported feeling misunderstood, which not only left them without appropriate support but also reinforced feelings of self-stigma, with some describing it as validating fears that they were “going mad.” An additional issue identified was the lack of continuity of care, which meant that individuals often saw multiple GPs, leading to inconsistent advice and conflicting explanations about their symptoms, which further contributed to confusion and frustration.

##### 3.1.3.2 Gaps in ophthalmology services

Participants described how ophthalmology services in the UK tend to focus on diagnosing the eye condition and providing information about the resulting visual impairment. Military veterans reported that while they often left the appointment well-informed about their diagnosed eye condition, they rarely received any information about the potential risk of developing CBS. As a result, many felt unprepared for the possibility of experiencing visual hallucinations. Participants noted that after receiving a Certificate of Visual Impairment (CVI), there was typically no further follow-up from ophthalmology services, especially for individuals with untreatable eye conditions (e.g., dry age-related macular degeneration). They also highlighted long waiting times between diagnosis of the eye condition/visual impairment and contact with vision rehabilitation officers, which raised concerns about individuals being left without information or support, particularly if they began experiencing hallucinations during this period. Some participants suggested that opticians could help bridge this gap. However, they acknowledged that not everyone attends regular optometry appointments and that awareness of CBS amongst optometrists and dispensing opticians can be inconsistent. Similarly, Eye Clinic Liaison Officers (ECLOs) were also seen as a potential source of information; however, participants reported inconsistent access to ECLOs (with some eye clinics lacking them altogether), as well as varying levels of CBS knowledge amongst those in the role.

##### 3.1.3.3 Sight loss charities bridging the gap

Participants noted that individuals who were referred to, or independently accessed sight loss charities (e.g., SSV, Blind Veterans UK) often had CBS symptoms identified by rehabilitation officers within these organizations. This was attributed to their knowledge of CBS and their proactive approach in initiating conversations and asking targeted questions, which helped take the onus off military veterans to disclose visual hallucinations themselves. Once CBS was suspected, rehabilitation officers typically encouraged veterans to consult their GP for a diagnosis and to rule out other potential diagnoses or complications (i.e., side effects from medications). Participants also noted that some sight loss charities run information sessions on CBS and organize peer-support events to encourage military veterans to share their experiences with others.

In summary, participants described a widespread lack of awareness of CBS across multiple areas of healthcare, including general practice and ophthalmology services. Although healthcare professionals such as ophthalmologists, optometrists and ECLOs were seen as being well placed to provide information about CBS, limited knowledge often meant that military veterans were not informed about the potential risk of visual hallucinations, leaving them unprepared for these experiences. As a result, sight loss charities often became the first point at which CBS was identified within military veterans. Together, these findings highlight a clear gap in healthcare services and provisions, suggesting a need for greater awareness of CBS amongst healthcare professionals, as well as a more consistent approach to identifying and supporting CBS in military veterans with visual impairments.

### 3.2 Development of a toolkit

The second aim of Phase 1 was to develop a toolkit to support military veterans and healthcare professionals in managing CBS and accessing healthcare services and provisions. Suggestions for what this toolkit should look like were gathered, and these would be used to inform the development of the toolkit. The parameters of the toolkit were defined by military veterans and healthcare professionals, and from this, two main themes were identified. The first theme, “The focus of the toolkit,” captures the main aim and audience for the toolkit, as well as the content that it needs to cover. The second theme, “Required features of the toolkit,” highlights participants’ preferences on how the information should be delivered, including preferred formats, accessibility considerations, and content features.

#### 3.2.1 The focus of the toolkit

Participants highlighted a clear need for the toolkit to prioritize raising awareness and improving knowledge of CBS amongst healthcare professionals, military veterans, and their families/carers. They emphasized the importance of developing a range of educational resources tailored to the specific needs of each group. For healthcare professionals, participants called for a general resource that could be shared across a range of healthcare services, including (but not exclusively) healthcare settings (e.g., GPs, Accident & Emergency staff), ophthalmology services (e.g., ophthalmologists, optometrists, dispensing opticians, ECLOs), mental health services, elderly care services (e.g., dementia teams), and rehabilitation services (e.g., vision rehabilitation officers). They recommended that the resource should cover key information explaining what CBS is, why it occurs, how to identify and diagnose it, and how to support its management both clinically and through self-management strategies. Participants also requested that a resource specifically for military veterans be provided, either at the point of diagnosis of a visual impairment or at a follow-up appointment within 3–6 months post-diagnosis. They advised that this resource should provide a more accessible summary explaining what CBS is, why it happens, and how it would affect them. It should also include a variety of practical strategies that can be used to help clear visual hallucinations, reduce the likelihood of their onset, and manage any potential psychological and emotional distress. Participants also requested guidance on the next steps in the process and details on whom the veteran can contact for support. In addition, military veterans also requested resources they could share with family members and carers to help them understand CBS and its causes, along with guidance on how to offer support, and information on next steps and where to access further support.

#### 3.2.2 Required features of the toolkit

For the military veterans’ resource, participants expressed a strong preference for audio resources, as visual impairments can often make reading difficult. In particular, formats such as Talking Books, USB sticks, and podcasts were recommended, and many military veterans were already familiar with these. Participants advised keeping audio resources brief, ideally 10–15 min, as this length provided sufficient information without causing boredom or loss of concentration. Regarding language, a direct and to-the-point approach was preferred, with personalized phrasing such as “let us talk about how it affects you” or “you have just been diagnosed” to make the content more relatable. For the resource to be accessible to individuals with hearing impairments, participants recommended using a narrator with a deeper tone. Participants wanted the resource to include statistical facts (e.g., 1 in 10 people) and concise information presented in a way that would be understandable to a lay audience. They also noted that recapping important information throughout the resource would help support memory retention. In addition, it was suggested that the resource should also include personal stories of military veterans’ experiences of visual hallucinations to help others understand what these experiences are like and recognize them more easily. The format of the resources for healthcare professionals and family members/carers was more flexible, and participants had no preference for either audio or paper-based resources, as they could see benefits in both approaches. It was more important to the participants that the resources were available to access online. There were also suggestions to include visual images in paper-based or electronic materials to illustrate examples of common visual hallucinations reported by military veterans to aid understanding of what CBS-related hallucinations can look like.

In summary, participants suggested that the main aim of the toolkit should be to raise awareness and knowledge of CBS amongst three distinct audiences: healthcare professionals, military veterans, and family members/carers. It was recommended that each audience member needs to know (as a minimum) what CBS is, how to manage it, and the next steps involved in getting a diagnosis and avenues for additional support. Additionally, participants stressed that resources for military veterans should prioritize accessibility, both in format (i.e., to account for visual and auditory impairments) and content (e.g., clear layperson language). For healthcare professionals and family members/carers, online accessibility was the most important feature. Together, these findings provide the initial parameters necessary to develop a toolkit that supports military veterans and healthcare professionals in managing CBS and accessing healthcare services and provisions.

### 3.3 Initial development of the toolkit

Considering the experiences, perspectives and the areas of need identified by both military veterans and healthcare professionals, it was decided that a psychoeducational toolkit should be developed, which should include a variety of resources that are tailored toward different audiences (e.g., healthcare professionals, military veterans, family members/carers), and that auditory formats should be prioritized. Such resources should include information on CBS and hallucinatory experiences, advice on management strategies, and guidance on healthcare services and available provisions. It was also important for resources to have an accessible language and contain information that was easy to remember. As a result, the toolkit was initially developed to include the following three resources.

Resource 1: Podcast for healthcare professionals—A podcast, approximately 40 min in length, was developed for individuals working within a variety of healthcare settings (e.g., general healthcare, ophthalmology, mental health services, elderly care services) or in vision impairment support roles (e.g., rehabilitation officers, support workers). The podcast is designed to educate healthcare professionals about CBS in military veterans with visual impairments. It covers the history of CBS, etiology and risk factors, diagnosis criteria, prevalence of CBS, clinical features and psychosocial impacts. It also includes examples of veterans’ experiences (drawn from the Phase 1 workshop), offers guidance on management from both self-management and clinical perspectives, and provides details of additional support services available for referral. The podcast and associated script can be used to obtain CPD (continuing professional development) credits for NHS healthcare professionals. The podcast will be available for free on various platforms, including Spotify and Apple Podcasts.

Resource 2: Podcast for military veterans and family members/caregivers—This 20-min (approximately) podcast was developed for military veterans with visual impairments and their family members/caregivers. The podcast explains in clear, layman-friendly language what CBS is, why hallucinations happen, and briefly discusses prevalence rates and risk factors. It also includes personal stories from military veterans (taken from the Phase 1 workshop), explores potential triggers and offers a variety of strategies for managing hallucinations. As part of this, an easy-to-remember acronym was introduced to help recall the strategies to implement during a visual hallucination episode (see Resource 3). The podcast also includes guidance on accessing healthcare and signposting to available support services and resources. The podcast will also be available for free on various platforms, including Spotify and Apple Podcasts.

**FIGURE 2:**
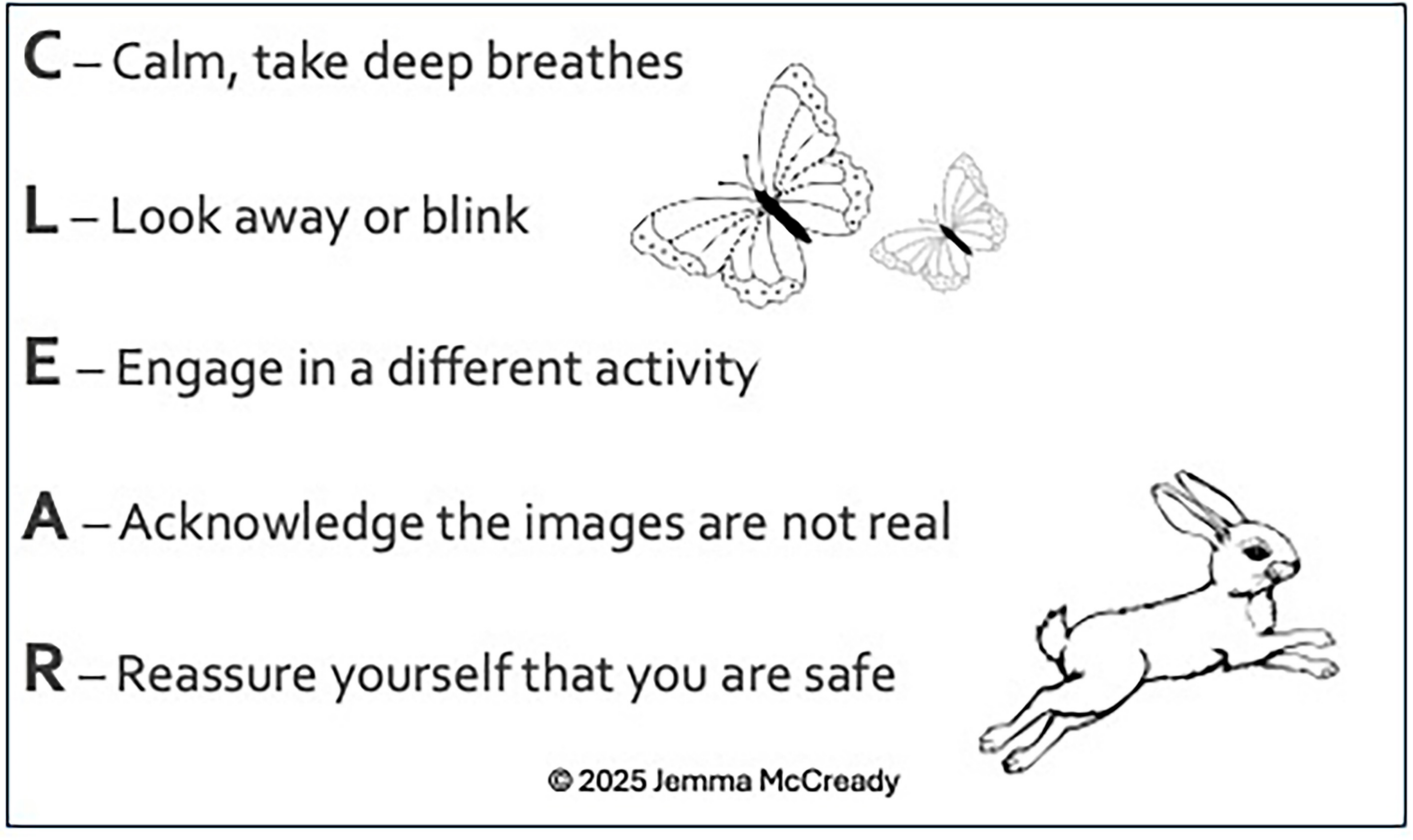
The CLEAR acronym, which is designed for military veterans to help “clear” CBS-related visual hallucinations.

Resource 3: CLEAR strategy for self-management of visual hallucinations—The CLEAR acronym was developed in response to the need for having a simple and easy way for military veterans to remember what to do if they experience a visual hallucination (Figure 2). The steps were devised based on the current literature surrounding CBS and anecdotal evidence provided by military veterans in the Phase 1 workshop. The recommended steps are designed to promote self-regulation, emotional reassurance and a practical response to help interrupt or reduce the impact of the visual hallucination in the moment. The CLEAR acronym was incorporated into both the veteran and healthcare professional podcasts (Resources 1 and 2) to ensure a consistent message, whereby the healthcare professional is reinforcing the same guidance given to the military veteran directly; this enhances the likelihood of the veteran remembering and implementing the steps during a visual hallucination.

### 3.4 Phase 2 findings

The aim of Phase 2 was to gather stakeholder perspectives and feedback on the initial toolkit developed (Resources 1–3) and to implement recommended alterations. During the Phase 2 workshop, military veterans and healthcare professionals listened to both podcasts and shared their initial perceptions of the toolkit, along with suggestions for improvement. See Supplementary material for the supporting quotes for each theme.

#### 3.4.1 Initial perceptions of toolkit

Initial perceptions of the resources included in the toolkit were highly positive, and participants appreciated having resources tailored to different audiences. The podcast designed for military veterans was described as informative and engaging, with several veterans saying they wished the resource had been available to them in the beginning when they first started experiencing visual hallucinations. They further explained that having that information and knowledge would have helped to alleviate some of the initial anxiety and fear surrounding what was happening, as they would have known straight away that it was CBS. Participants thought the resource was well-structured and appreciated that it provided sufficient information without being overly in-depth or burdensome. Military veterans appreciated having sections of information broken up and thought the jingle was effective, as it gave them a moment to process what had been said and recognize that the topic was moving on. The CLEAR strategy was also well received by both military veterans and healthcare professionals. For military veterans, it was described as an easy way to remember what they needed to do in the moment to get rid of the visual hallucination. Healthcare professionals particularly liked it because it provided an easier way to explain management strategies to military veterans, and they thought it was simple enough for them to remember. Participants also thought the podcast was suitable for family members/carers, and there was sufficient content for them to understand and support the individual. Regarding the podcast for healthcare professionals, participants described it as a good resource that contained the main information needed to recognize, diagnose, and support military veterans with CBS. They appreciated its broad applicability, noting that it was general enough to be useful for professionals across various healthcare services. In addition, they appreciated that the CLEAR strategy was also included in the healthcare professionals’ resource, as it provided a sense of consistency in the approach to managing visual hallucinations.

#### 3.4.2 Suggestions for improvements

One of the key suggestions from participants was to create an additional, shorter podcast for military veterans. They felt that a short 3–5-min version would be beneficial, as it would allow people, especially military veterans, to quickly access key information with the option to explore more detailed resources later. Healthcare professionals also expressed a desire to have printed resources available for military veterans to take home during community visits. For the healthcare professionals’ resource, participants recommended including additional information on the etiology of CBS, potential triggers for hallucinations (e.g., stress), and practical guidance on screening for medication side effects. Participants also emphasized the need for the toolkit to be disseminated more widely. They suggested running social media campaigns on platforms like YouTube, Instagram and TikTok to expand their reach and accessibility. They also suggested developing some visual materials that could be used on these platforms, such as short videos or digital images illustrating common CBS-related visual hallucinations. Participants also expressed a need for more personalized, in-person resources, such as psychological therapies (e.g., emotion-focused therapies, cognitive behavioral therapy), to support military veterans experiencing high levels of emotional and psychological distress because of the visual hallucinations.

Overall, participants provided positive feedback on all three resources, describing them as well-aligned with the priorities and needs discussed in the first workshop. The resources were considered informative and covered the main information that healthcare professionals, military veterans, and family members/carers needed to understand CBS. Participants also appreciated the CLEAR strategy, finding it to be a memorable and straightforward approach for military veterans to follow practical steps for managing visual hallucinations, as well as for healthcare professionals to recommend as part of their guidance. Key areas for improvement included requests for a shorter podcast to facilitate quicker access to essential information, printed materials for healthcare professionals to share with military veterans, and the inclusion of additional information in the healthcare professionals’ resource. Participants also recommended expanding the toolkit’s reach by creating visual materials, such as short videos and digital images, to be shared on social media platforms like TikTok, YouTube and Instagram.

### 3.5 Refinement of the toolkit

In response to participant feedback, several alterations and additions were made to the initial toolkit. As such, only changes that were feasible and aligned with the initial purpose of the toolkit, as identified by participants in Phase 1 (i.e., as a psychoeducational toolkit), were implemented. These included the addition of a shorter podcast for military veterans and their family members/carers, the inclusion of additional content within the healthcare professionals’ podcast, and the development of a short, animated video designed for general audiences to be disseminated on social media platforms.

Resource 4: Short podcast for military veterans and family members/carers—A 5-min (approximately) podcast was developed in response to requests for a shorter resource for military veterans and family members/carers (Supplementary material). This podcast covers the same information as Resource 2 but presents it in a more concise and less detailed format. It focuses on explaining why CBS occurs, what hallucinations might look like, how to manage them using the CLEAR strategy, and where to find further information and support. This podcast is intended as a quick, introductory guide, and people are encouraged to seek out more detailed resources if needed. The podcast will be available for free on both Spotify and Apple Podcasts.

Resource 5: Animated video for general audiences—A 3-min (approx.) animated video was developed for general audiences to raise awareness of CBS in military veterans with visual impairments (Supplementary material). The video employs a whiteboard animation style, a technique that utilizes hand-drawn illustrations, often on a whiteboard, to explain concepts visually. The animation is accompanied by a voice-over narration that explains what CBS is, how it presents, the impacts on military veterans, how to manage visual hallucinations (using the CLEAR strategy), and next steps for seeking support. The animated video will be disseminated on YouTube and TikTok to maximize accessibility and reach.

In summary, the final toolkit comprises a range of accessible, practical resources, including targeted podcasts for healthcare professionals (Resource 1) and military veterans and their families (Resources 2 and 4), the CLEAR self-management strategy (Resource 3), and a short, animated awareness video for the general public (Resource 5). Together, these resources provide practical information tailored to the specific needs identified by military veterans and healthcare professionals, with the overall aim of enhancing awareness, recognition, and management of CBS in military veterans.

## 4 Discussion

This study examined the experiences of living with and managing CBS, as well as accessing healthcare services and resources, from the perspectives of military veterans and healthcare professionals. From these experiences, a psychoeducational toolkit, comprising five resources, was developed and further refined through discussions with military veterans, CBS, and healthcare professionals. The final toolkit included three podcasts, two for military veterans and their families/carers and one for healthcare professionals (which can be used for CPD credits). Additionally, it featured an animated video for general audiences and a practical strategy for managing visual hallucinations (CLEAR).

One of the key issues identified was the significant distress experienced by military veterans, particularly at the onset of CBS. Many veterans reported receiving no information about CBS at the time of their diagnosis with a visual impairment, leaving them unprepared for the possibility of visual hallucinations. This lack of forewarning contributed to heightened fear and anxiety when hallucinations first occurred. Many military veterans reported questioning both their physical and mental health, with prominent fears of cognitive decline or neurological illness—sentiments echoed in previous research (Dave et al., 2024; Jurisic et al., 2018). These health-related anxieties contributed to feelings of stigma, which discouraged many military veterans from disclosing their symptoms to family members and healthcare professionals. This reluctance to disclose experiences of visual hallucinations aligns with previous studies showing how military veterans often avoid discussing CBS due to fears of being misdiagnosed with psychiatric conditions such as dementia, psychosis or schizophrenia (Dave et al., 2024; Jones et al., 2025b). Our findings further revealed that even when military veterans did disclose their experiences, responses from family members and healthcare professionals frequently reinforced these fears, with symptoms often dismissed or misattributed to cognitive deterioration. Such misinterpretations led to inappropriate referrals and, in some cases, delayed access to appropriate support services, amplifying emotional distress. Addressing this widespread lack of awareness was identified by participants as the main priority for the toolkit. They strongly advocated for targeted education to improve understanding amongst military veterans, their family members, and healthcare providers, to normalize CBS, reduce stigma, and facilitate access to appropriate healthcare services and provisions.

To address this need, the toolkit included two podcasts for military veterans and their families/carers, along with a short, animated video designed for broader audiences. These resources provide information about CBS, including its causes, symptoms, potential triggers, as well as practical strategies for managing visual hallucinations and guidance on where to seek support. These resources are designed to be shared with military veterans at the point of diagnosis with a visual impairment. Family members and caregivers can also utilize these resources to gain a deeper understanding of CBS and provide informed support. The five-minute podcast provides a brief introduction to CBS. At the same time, the 15-min version offers a more in-depth explanation for those seeking additional information, such as when a military veteran begins to experience visual hallucinations. The animated video was developed for a broader audience, including family members/carers, healthcare professionals, and the general public, as it provides a generic overview of CBS, designed to raise broader awareness of CBS. Together, these resources aim not only to educate but also to validate the experiences of military veterans, reduce stigma, and promote early disclosure and support-seeking behaviors. Similar priorities have been identified by other military veteran groups, healthcare practitioners, and researchers, highlighting a broader systemic unmet need for educational resources on CBS (Dave et al., 2024; Jones et al., 2021a). This suggests that the resources developed in this study may have broader applicability and utility beyond the initial veteran cohort in which they were developed.

In addition to the need for improved awareness, participants in this study highlighted significant gaps in practical strategies to manage CBS-related visual hallucinations. Military veterans frequently described relying on personal, trial-and-error approaches to manage visual hallucinations, often in the absence of any formal guidance. Commonly used techniques included blinking, moving their head or eyes, changing the environment (such as adjusting lighting), or using distraction techniques like listening to music. While some individuals reported occasional success, others found these strategies inconsistent or ineffective, particularly in situations where hallucinations were persistent or matched the surrounding environment. Our findings align with those of previous studies, in which individuals with CBS similarly reported using behavioral techniques, including rapid blinking, environmental adjustments, and distraction strategies (Christoph et al., 2024; Dave et al., 2024). However, some literature highlights a lack of formal evidence on the efficacy of these techniques, with many studies also reporting inconsistent effectiveness amongst individuals using them (Dave et al., 2024; Jones et al., 2025b). Alongside strategies to interrupt hallucinations, some military veterans described learning to recognize personal triggers and taking preventive steps to minimize the onset of hallucinations. For instance, stress and tiredness were reported as common triggers, and some participants implemented strategies such as pacing daily activities, taking rest breaks, or engaging in relaxing and distracting activities, like watching TV, to help minimize the onset. These observations are consistent with existing research, which suggests that stress and fatigue commonly exacerbate CBS symptoms (Jones et al., 2021b; Vukicevic and Fitzmaurice, 2008). In the broader literature, strategies such as pacing and taking rest breaks are routinely employed to manage fatigue (Casson et al., 2023), which may help explain why military veterans found these approaches beneficial. Healthcare professionals also noted that mindfulness exercises were a helpful tool for some military veterans. Although mindfulness has not been formally evaluated in CBS populations, a pilot study assessing the feasibility of a mindfulness intervention for individuals experiencing auditory hallucinations reported small to moderate reductions in distress and disruption caused by hallucinations (Louise et al., 2019). It is thought that mindfulness practices may help reduce the emotional impact of hallucinatory experiences more generally by improving present-moment awareness and reducing the tendency to engage with distressing hallucination content (Louise et al., 2019). Although anecdotal evidence suggests that mindfulness and identifying and controlling personal triggers may be helpful, the effectiveness of such strategies for managing CBS-related visual hallucinations requires further investigation.

In this study, both military veterans and healthcare professionals emphasized the need for guidance to support the management of CBS-related visual hallucinations. To address this gap, the CLEAR acronym was developed as a simple, step-by-step tool to guide military veterans through practical actions they could implement during a visual hallucination. The use of acronyms is a well-established technique in public health communications and campaigns, commonly used to simplify complex information, enhance memory retention, and facilitate rapid recall of key actions under pressure (Hagberg et al., 2024). For example, the widely recognized Act FAST campaign, which uses the acronym FAST (Face, Arms, Speech, Time) for recognizing and acting upon symptoms of Stroke (NHS England, 2020), has been shown to improve public knowledge, increase symptom awareness and encourage timely help-seeking behaviors (Rasura et al., 2014). Evidence also suggests such campaigns can promote long-term information retention and increase proactive health behaviors, such as seeking medical advice (Flynn et al., 2014). Drawing on these principles, the CLEAR strategy incorporates the informal techniques that military veterans were already using into a structured framework to improve memory recall and build confidence in self-managing visual hallucinations. As such, initial feedback from military veterans and healthcare professionals was positive, and veterans described CLEAR as an easy-to-remember approach that provided reassurance of what to do in the moment to manage a visual hallucination. Healthcare professionals welcomed the strategy as a practical tool to simplify explanations during consultations and to deliver consistent advice to military veterans. Upon reflection, it is anticipated that the CLEAR strategy may offer the greatest value to individuals in the early stages of CBS by providing timely guidance that could help reduce distress at the onset of hallucinations. However, since participants in this study only provided perceptions of the strategy without formally testing it in practice, further research is needed to evaluate its real-world acceptability and effectiveness. For instance, a pilot study should explore how military veterans would use the CLEAR strategy in everyday settings, assess its impact on managing hallucination-related distress, and gather feedback from healthcare professionals delivering the strategy in routine clinical care.

In addition to the challenges of managing CBS symptoms, participants described significant barriers in accessing appropriate healthcare services and provisions. A key concern raised by both military veterans and healthcare professionals was the widespread lack of awareness of CBS, particularly amongst general practitioners and professionals working in ophthalmology services. This finding is consistent with existing literature, which shows that a high proportion of healthcare professionals remain unaware of CBS, even within relevant clinical specialities (Jones et al., 2021a). Participants highlighted the importance of receiving information about CBS at the point of diagnosis of a visual impairment, noting that earlier forewarning could have reduced the emotional distress experienced when visual hallucinations began. Instead, many military veterans reported first learning about CBS through non-clinical routes, such as vision loss charities or from conversations with other military veterans, rather than from healthcare professionals. While based on a small number of accounts, these experiences suggest a broader implementation gap in clinical practice. Although consensus clinical management guidelines (O’Brien et al., 2020) recommend proactive patient education, forewarning, and reassurance, our findings indicate that such practices were not consistently applied in routine care. This gap between guidelines and practice is likely driven, at least in part, by limited clinician awareness and education about CBS. In response to this gap, the toolkit developed in this study includes educational resources for healthcare professionals. The 40-min podcast and accompanying script provide an overview of CBS, including its prevalence, etiology, diagnostic criteria, key clinical and psychosocial features, and guidance on clinical and self-management strategies. To promote consistent messaging, the CLEAR strategy for self-management of visual hallucinations is also introduced within the podcast. Embedding this tool across both veteran-facing and clinician-facing resources helps ensure that healthcare professionals are equipped to reinforce the same practical advice given directly to veterans.

To maximize the impact of these resources, it is essential that they reach the healthcare professionals most likely to engage with patients in the early stages of visual impairment, when timely information about CBS can be most effective. Participants identified ECLOs as the preferred first point of contact for delivering information about CBS, given their supportive role within eye clinics and regular interaction with individuals adjusting to visual impairment. However, participants noted inconsistencies in access to ECLOs, highlighting both the absence of the role in some eye clinics and the limited knowledge and awareness of CBS among those in the role. This observation aligns with national service evaluations, which show that only one-third of the busiest NHS trusts in England (37.3%; 56 out of 150) have an ECLO embedded within their eye clinics, leaving over 60% of eye clinics without this provision (Papastefanou et al., 2020). Moreover, even when ECLO support is available, access is often delayed, with data indicating that nearly 70% of patients do not see an ECLO until around 13–18 months post diagnosis with a visual impairment (Papastefanou et al., 2020).

Furthermore, evaluations of ECLO caseloads suggest that discussions about CBS or the risk of visual hallucinations are rarely documented, indicating that such conversations may not be a routine part of these appointments (Menon et al., 2020). Given the limited and often delayed access to ECLOs, it is equally important to educate other frontline healthcare professionals, including GPs, optometrists, and dispensing opticians. The broader dissemination of the toolkit across these roles could help address the current gaps in healthcare services and provisions, ensuring that more military veterans receive timely and appropriate support. As a next step, efforts should focus on integrating the toolkit into existing clinical workflows, such as through digital healthcare platforms (e.g., NHS eLearning4health, CPD portals), and partnerships with high-street optometry providers.

Another key area of discussion centered on the emotional and psychological responses to CBS-related visual hallucinations. The variations in reactions to the hallucinations were interesting, as a lot of the military veterans within this study did not feel distressed in response to the content of their visual hallucinations. The reason for this could be twofold. First, many of the military veterans reported experiencing more neutral or non-threatening visions and were able to quickly identify the hallucination as not being real. This aligns with previous research showing that neutral or positive hallucinations are generally linked to lower emotional and psychological distress, whereas threatening or disturbing content tends to provoke stronger adverse reactions (Dave et al., 2024; Menon, 2005). Secondly, the military veterans in this study were all receiving support for vision-related services, which likely included prior education about CBS, along with reassurance and practical support for managing visual hallucinations. This may have contributed to their relatively low levels of distress. Indeed, existing research supports this view, showing that individuals who are informed about CBS, particularly around the underlying cause, tend to experience less emotional distress and feel more able to manage hallucinations (Cox and Ffytche, 2014; Dave et al., 2024). However, this was not a universal experience as healthcare professionals noted that military veterans outside of formal support networks often experienced emotional distress in response to their visual hallucinations, particularly when the hallucinations contained harmful or threatening images (e.g., tigers), or when the content of the vision matched the surrounding environment, for instance, a vision of a train while at a train station, or visions of people and cyclists while crossing a road. These hallucinations posed significant challenges, as military veterans found them much harder to recognize and often reacted as if they were real (e.g., attempting to board a non-existent train, or waiting for non-existent pedestrians to pass). Similar difficulties were reported by Fisher et al. (2023), who described six case studies in which individuals experiencing such hallucinations reacted physically, such as stepping aside to avoid perceived obstacles, striking out, or stopping in fear, which in some instances led to falls and serious injury. Concerns were raised about how to manage these types of hallucinations, particularly around how to help military veterans recognize the hallucination more quickly to reduce the risk of falls and other severe consequences. While this specific request could not be fully addressed in the toolkit due to limited evidence on managing these experiences, brief advice was included for both military veterans and healthcare professionals, focusing on communication with healthcare providers and referrals to services such as fall prevention teams. However, further research is needed to explore these experiences and identify ways to support individuals, as previous studies also report this to be a common experience among military veterans (Christoph et al., 2024; Fisher et al., 2023). It may be that veterans experiencing high levels of distress, or hallucinations that closely match real-world environments, could benefit from one-on-one psychological support, such as emotion-focused therapies or cognitive behavioral therapy, to help manage their reactions and reduce emotional distress.

### 4.1 Strengths and limitations

A key strength of this study is its co-production approach, which placed the lived experiences of military veterans and perspectives of healthcare professionals at the center of the research process. This approach allowed the direction, design and content of the toolkit to be directly informed by those who have firsthand experience of CBS, thus enhancing the ecological validity of the toolkit. However, this study has some limitations. First, our sample consisted only of visually impaired military veterans from a specific UK-based vision loss charity, who were already receiving specialist care and gold-standard rehabilitation support, which is not available to all those with CBS (e.g., those who do not meet the criteria to be charity beneficiaries). As such, our participants may have very different experiences and coping strategies in comparison to other visually impaired military veterans who are not engaged with such support services. This limits the generalizability of the findings to the broader veteran population.

Additionally, all military veterans and healthcare professionals reflected specifically on their experiences within NHS Scotland services and provisions. Given the known structural and operational differences between healthcare systems across the UK, these findings may not fully reflect the experiences of military veterans accessing healthcare services in England, Wales or Northern Ireland. Moreover, military veterans are often considered to possess greater psychological resilience and atypical life experiences, which may diminish their fear responses.^1^ As a result, CBS may be perceived as more distressing by non-veteran populations, and any generalization of these findings should therefore be approached with caution.

Finally, although the development of the toolkit was grounded in qualitative insights and co-production principles, its effectiveness in real-world settings has yet to be formally evaluated. The current study focused on the design and refinement of the resources based on stakeholder input rather than outcome measurement. As such, we cannot draw conclusions on the toolkit’s impact on clinical outcomes, symptom management, or help-seeking behaviors. Further research is necessary to explore this aspect in more detail.

### 4.2 Future research

Future research should prioritize the formal evaluation of the psychoeducational toolkit developed in this study. Specifically, a pilot study is needed to explore military veterans’ engagement with the toolkit and assess its acceptability, usability, and potential to support earlier disclosure, reduce emotional and psychological distress, and improve confidence in managing CBS-related visual hallucinations. Further evaluation is also warranted to examine the toolkit’s relevance and practical value for healthcare professionals, including its utility in clinical consultations and its ability to improve knowledge and confidence in recognizing and managing CBS. Subsequent work would need to disseminate the toolkit across healthcare services and vision-related services, as well as within military veteran communities. An additional area for future research involves evaluating both current and potential management strategies. For instance, military veterans experiencing high levels of emotional distress or complex hallucinations may benefit from tailored intensive psychological support in the form of intensive treatment rather than traditional out-patient, 50-min, weekly sessions. Therefore, research exploring the feasibility and acceptability of interventions such as cognitive behavioral therapy, emotion-focused therapy, or mindfulness-based approaches could potentially be beneficial for managing emotional distress and improving coping amongst military veterans with CBS.

## 5 Conclusion

This study explored the experiences of living with and managing CBS, as well as accessing healthcare services and provisions, from the perspectives of military veterans and healthcare professionals. These insights informed the co-production of a psychoeducational toolkit comprising five resources, which was iteratively developed and refined in collaboration with both groups. The toolkit was designed to address the widespread lack of awareness, reduce emotional distress, and provide practical guidance for managing CBS-related visual hallucinations from both a clinical and self-management perspective. Future research should focus on evaluating the effectiveness and utility of the toolkit, as well as exploring its integration into clinical care pathways and military veteran support networks, to maximize reach and impact across key healthcare professional groups and other military veteran communities.

## Data Availability

The raw data supporting the conclusions of this article will be made available by the authors, without undue reservation. Requests can be directed to derek.farrell@northumbria.ac.uk.

## Footnotes

1
https://journal-veterans-studies.org/articles/10.21061/jvs.v9i1.405
