# Supplementary material for: Understanding healthcare accessibility for military veterans living with Charles Bonnet Syndrome: co-production of a psychoeducational toolkit
Source: Front Psychol. 2026 Jun 3;17:1698432. doi: 10.3389/fpsyg.2026.1698432 (PMC13273905; doi:10.3389/fpsyg.2026.1698432)
Supplement: Supplementary file 1 [file Table_1.docx]

Supplementary Material

# Podcast script and link for healthcare professionals

**Link to podcast: ….**

Insert music introduction

In the quiet of night, through the kitchen pane,
A tiger appears, wild eyes filled with pain.
A warrior once strong, now battles unseen,
Where shadows of war mix with visions obscene.

The stripes in the window, the growl in the air,
A beast in the glass, but no one is there.
Memories of battles, of courage, of strife,
Now merge with hallucinations that cut like a knife.

His hands, once steady, now tremble with fear,
As he stares at the tiger, so crystal, so clear.
But the kitchen is still, the room’s filled with calm,
Yet the beast lingers on, like a troubling psalm.

A soldier, a man, now lost in the fight,
Not with guns or bombs, but with visions at night.
In his mind’s foggy war, he still wears his shield,
Hoping for peace, that the tiger will yield.

(A UK Military Veteran’s Experience of Charles Bonnet Syndrome)

Hello and welcome. My name is Jane Arnfield, and I am from Northumbria University, Newcastle. Today, we discuss an important health issue called Charles Bonnet Syndrome – or CBS for short. Charles Bonnet Syndrome (CBS) is a condition that affects some individuals when they lose all or part of their vision. It results in visual hallucinations, which are experiences of seeing things that do not actually exist. These hallucinations can be simple (flashes of light or shapes) or, as the example at the start highlighted, can be more complex (detailed images of people, animals, or objects).

So, what Causes Charles Bonnet Syndrome? With healthy vision, light enters the eye and is received by the retina (the light-sensitive tissue at the back of the eye). The retina converts these light rays into visual messages, which are sent to the brain, allowing us to see. When individuals lose vision from conditions like age-related macular degeneration, glaucoma, or diabetic retinopathy, their visual system fails to process new images. Without visual data coming in through the eyes, the brain fills the void by creating or recalling stored images for you to perceive. This phenomenon leads to the visual hallucinations associated with CBS. It is very similar to how individuals who have lost a limb may experience phantom pain and does not indicate a mental health issue.

CBS was named after the individual who first analysed and described visual hallucinations in psychologically normal individuals. This podcast aims to explain CBS and how to better understand it, explore practical considerations for managing it, and outline additional resources for seeking further support.

By means of background, this podcast is a product of a research project commissioned by the Office of Veterans Affairs to investigate the lived experience of military veterans who experience CBS. The primary objective is to raise awareness of CBS within the NHS workforce.

**Section 1: Enhancing Understanding of Charles Bonnet Syndrome (CBS)**

So, in more detail, what exactly is Charles Bonnet Syndrome? At this point, a more critical question might be, ‘Who on earth is Charles Bonnet?’ Charles Bonnet (1720-1793) was an 18^th^ century Swiss natural scientist and philosopher born into a wealthy family in Geneva. At the age of seven, Bonnet developed partial hearing loss^[[1]](#footnote-1)^. This may partially explain his interest in sensory deprivation. Later, when in his early 20s, Bonnet encountered intense eye pain accompanied by a gradual loss of vision. As he could no longer use a microscope to see things in fine detail, he was forced to abandon his research in the field of natural sciences and shift toward more philosophical and theoretical domains. By the time Bonnet reached 40 years of age, he was severely visually impaired. A research article published in 2006 highlighted that Bonnet himself developed visual hallucinations, including visual impairment, lack of cognitive deficiencies, and a tendency for these episodes to occur in a quiet and reflective setting. CBS’s distinct feature of complex visual hallucinations is different from either a psychiatric disorder or a type of dementia. There is no underlying pathological abnormality besides visual degeneration, with no cognitive deficiency or evidence of degenerative disease.

In Bonnet’s^[[2]](#footnote-2)^ essay published in 1760, he describes the experiences of his grandfather and the problems he encountered:

“I should tell about a strange case that would be considered fabulous if not supported by testimonies of the highest credibility. However, the release of this psychological phenomenon would deserve a writing of its own. I will simply say that I know a respectable man full of health, of ingenuousness, judgement, and memory, who, completely alert and independently from all outside influences, sees from time to time, in front of him, figures of men, of women, of birds, of carriages, of buildings … etc. He sees these figures make various movements: getting closer, going away, fleeing, diminishing or increasing in size, appearing or disappearing; he sees the buildings rise in front of his eyes and a display of all the outside construction material. The tapestries in his apartment appear to change suddenly; these tapestries cover themselves with painting displaying different landscapes. Another day, instead of the tapestries and furniture it is only the naked walls with an assembly of raw materials. All these visions appear to him in perfect clarity and affect him as strongly as if the objects themselves were present. However, these are only paintings because the men and women do not talk and no noise comes to his ear. All of this appears to have its seat in the part of the brain that commands the sense of sight. The person I am talking about was subject at different times and at an advanced age to cataract operations on the two eyes. The great success of this operation would probably never have been challenged if a less ardent desire to read had enabled this elder to better manage his sight as it deserved.” (Bonnet 1760 ).

The term CBS was later coined by the French neurologist Georges de Morsier^[[3]](#footnote-3)^ in a publication in 1967 titled, ‘*Visual hallucinations in the aged without mental deficiency’*. However, it has only become more known in the English-speaking medical community since the early 1980s. The World Health Organisation, in its latest edition of the International Classification of Diseases (ICD-11), formally recognised Charles Bonnet syndrome as a distinct clinical condition (Code: 9D56). It is described as follows:

“…. the experience of complex visual hallucinations in a person who has experienced partial or complete loss of vision. Hallucinations are exclusively visual, usually temporary, and unrelated to mental and behavioural disorders.

Although relatively common, Charles Bonnet Syndrome still gets very little attention from doctors and healthcare professionals.

Concerning the prevalence of CBS, a meta-analysis of eleven studies which included over 4000 individuals with visual impairment found that approximately just under 20% of patients aged 40 and above experienced CBS. Considering that an estimated 239 million individuals have moderate visual impairment or worse, it is estimated that the global number of patients with CBS is just over 47 million^[[4]](#footnote-4)^.

In a further study^[[5]](#footnote-5)^, 26% of patients with low vision experienced CBS. There was no significant difference between men and women concerning who was affected. It often took time for patients to realise that the images they were seeing as authentic were not, in fact, real. Simple hallucinations, such as patterns, occurred as frequently as more complex ones, like images of people, body parts, or faces. Research suggests that the most common hallucinations involve animals. Most visual hallucinations lasted only a few seconds and were more likely to occur during the day and in bright settings. All patients saw these images with their eyes open, and the hallucinations generally didn’t move when they looked around. Importantly, what this study highlighted was that most individuals with CBS didn’t discuss their hallucinations or seek help from a doctor, ophthalmologist, optician, or health professional. This has also been supported in other recently published studies, which highlight how many CBS patients were not informed about the benign nature of these hallucinations, and many had never shared their experience with anyone. These findings are important, as CBS is stressful for the patient and contributes to a lower quality of life.

Cultural factors may influence the reporting and recognition of CBS. In some cultures, visual hallucinations are stigmatised, leading individuals to conceal their experiences. This underreporting can result in lower observed prevalence rates in specific populations^[[6]](#footnote-6)^.

A study titled "‘They’re creepy creatures with human-like features’: children’s experiences of visual hallucinations in Charles Bonnet syndrome—a qualitative study" by Jones^[[7]](#footnote-7)^ et al., published in *Archives of Disease in Childhood* in January 2025, delves into the experiences of children aged 7 to 15 with inherited retinal diseases living with CBS, along with their parents. The key findings from this study highlighted that CBS in children causes considerable distress and psychological harm. The critical findings from this study focus on five areas: 1. Diagnosis journey – widespread lack of awareness of CBS, which in turn leads to significant frustration for parents and families; lack of awareness and understanding within the healthcare sector; an over-cautiousness by healthcare professional to formally diagnose CBS resulting in delays or inappropriate treatment interventions being offered. 2. Variations in visual hallucination phenomenology. 3. The impact of CBS on children, their parents and families. 4. Exploring better ways of managing visual hallucinations. 5. Experiences in accessing help and support. The conclusion from this study reads as follows:

“The journey towards understanding and managing CBS for both parents and children is challenging. Although coping strategies can lead to improved adjustment, visual hallucinations compound the difficulty of living with a chronic visual impairment. Healthcare providers are integral in ensuring patients and families are effectively supported to allay fears and promote psychological well-being.” (page 1)

A recent study carried out by Northumbria University, Newcastle, captured the experiences of military veterans who encounter CBS hallucinations. Results from this study captured the same experiences in military veterans as the previously mentioned research into CBS with children. Concerning the phenomenology of visual hallucination in CBS, a retired naval officer described one poignant example:

“I was out walking my dog once, and I could see two people approaching me. They were ladies, and they were dressed in Japanese kimono and they walked up to me until they were about 20, 30 feet away, and I could see the faces by that time, they were quite clear, and I just watched them, and then I said through my voice ‘Hello, can you see me?’, and they just vanished, they just vanished”.

A further example provided by a military veteran outlines potential risks with CBS:

“I could see bugs or insects crawling around in my breakfast cereal. I then couldn’t eat anything.”

This highlights how, for some, such CBS visual hallucinations can significantly impact people’s daily lives and negatively affect their self-care and daily activities. Visually impaired military veterans exhibit a notably higher prevalence of CBS compared to the general population. Regardless of hallucination content, CBS has a substantial impact on the lives of people with visual impairment^[[8]](#footnote-8)^.

[Brief Music Break here]

**Section 2: Practical Considerations**

In this next section, we will consider more practical considerations concerning CBS. First, we want to focus on how we can effectively manage CBS and help those affected live better, more comfortable lives. Currently, there are no established medical treatments for CBS. There are several approaches to supporting someone with CBS, so let’s examine them. These include:

1. CBS informed psychoeducation
2. Access to triage and risk assessment through CBS-informed General Practitioners
3. Enhancing affect regulation skills
4. Promoting social support and engagement
5. Behavioural exercises in managing the distress of visual hallucinations
6. Nurturing compassion-focussed self-talk/ language
7. Identifying stress triggers that may precipitate the onset of CBS visual hallucinations
8. Addressing and challenging stigma and prejudice
9. Triage and accessing evidence-based trauma treatment (including Trauma Focused-Cognitive Behavioural Therapy (TF-CBT) and Eye Movement Desensitisation and Reprocessing (EMDR))
10. Enhancing public and health care systems’ awareness of CBS across the lifespan and concerning cultural sensitivities

So, let’s expand on these ten themes in more detail.

The first aspect is Charles Bonnet Syndrome (CBS)-focused psychoeducation**.** This is essential for managing the condition, aiming to raise awareness and understanding among individuals with CBS, particularly military veterans, their caregivers, and healthcare workers. It involves educating people about the nature of CBS, clarifying that visual hallucinations are not signs of mental illness but rather results of significant vision loss, often due to conditions like macular degeneration or glaucoma. Psychoeducation helps normalise the experience of hallucinations, reducing the stigma and anxiety that can arise from such symptoms. Key points in CBS-focused psychoeducation include explaining the different types of hallucinations that can occur, such as seeing people, animals, or objects, and emphasising that these images are vivid yet not real. It also informs individuals that they are usually aware that what they see is not present, which helps them manage their reactions. By providing this information, psychoeducation enables individuals to understand their symptoms better, reduce distress, and encourage proactive coping strategies, ultimately improving their quality of life.

Military veterans with Charles Bonnet Syndrome (CBS) should be screened by a CBS-informed general practitioner (GP) who can effectively identify the condition, assess its severity, and conduct an appropriate risk assessment. This initial screening ensures that veterans receive the proper care and are triaged based on the urgency of their symptoms. Once assessed, veterans should be referred to CBS-informed ophthalmic specialists who can provide targeted interventions for their vision loss and related symptoms. Additionally, the GP or specialist should direct veterans to relevant support systems, such as mental health services, peer support groups, and community agencies, that are well-equipped to manage CBS's emotional and psychological aspects. This coordinated, multi-disciplinary approach ensures that veterans receive comprehensive, trauma-informed care that addresses both the physical and psychological impacts of CBS.

Enhancing affect regulation skills in military veterans with CBS is crucial for helping them manage the emotional and psychological challenges associated with vivid visual hallucinations. Veterans, often facing complex histories of trauma and visual impairment, may find these hallucinations distressing as they can trigger anxiety, fear, or frustration. Developing affect regulation skills, such as mindful breathing, meditation, yoga, breathwork, mindful-focussed exercises, and emotional reappraisal, can aid military veterans in better coping with these feelings when the hallucinations arise. These skills empower military veterans to regain control over their emotions and reactions, mitigating the impact of distressing experiences. For military veterans with post-traumatic stress disorder (PTSD), integrating these skills can assist in managing both trauma-related symptoms and the emotional ramifications of CBS. Additionally, enhancing affect regulation can bolster overall psychological resilience, improving mental health and a better quality of life for veterans coping with CBS.

Enhancing and promoting social support and engagement for military veterans with Charles CBS is vital for emotional well-being and recovery. Social connections provide military veterans with a sense of community and understanding, which can reduce feelings of isolation often experienced due to CBS. Encouraging military veterans to participate in peer support groups to share experiences and coping strategies fosters a supportive environment. Involving family members and caregivers in psychoeducation about CBS strengthens the veteran’s support network. Furthermore, engaging in social activities or veteran-focused events can offer a sense of purpose and help distract from distressing hallucinations. Building a strong social support system helps veterans feel more connected and understood, improving their overall quality of life.

Connecting with support groups and sight-loss charities can be particularly valuable. Charities such as Sight Scotland Veterans, Sight Scotland, the Royal National Institute of Blind People (RNIB), Blind Veterans UK, and Esme’s Umbrella provide support, reassurance, and practical advice for living with and managing CBS. Most of these charities also have helplines where you can speak with someone who can offer more tailored information and guidance on CBS and assist you further with your next steps.

Military veterans with CBS can benefit from various behavioural techniques to better manage their symptoms and experiences. One practical approach is cognitive-behavioural strategies, which assist veterans in reframing negative thoughts and beliefs about their hallucinations. By recognising that the hallucinations are not real and do not pose a threat, veterans can alleviate anxiety and fear. Grounding techniques, such as focusing on their senses or engaging in activities that keep them present, can also help manage overwhelming experiences. Behavioural activation, which involves maintaining a structured daily routine and staying engaged in meaningful activities, can decrease the likelihood of hallucinations triggered by boredom or isolation. These techniques empower veterans to take control of their responses, reducing distress and enhancing emotional regulation.

A further additional practical strategy uses the acronym **CLEAR** to manage visual hallucinations, and introduced the acronym **CLEAR**, which stands for **C – Stay calm**; **L – Look away or blink**; **E – Engage in a different activity**; **A – Acknowledge the images aren’t real**; and **R – Reassure yourself that you’re safe.**

Nurturing compassion-focused self-talk and language is particularly valuable for military veterans with CBS, as it helps them manage the emotional distress associated with their hallucinations through a lens of compassion. Veterans may experience feelings of confusion, fear, or frustration when confronted with vivid and often unsettling visions, and negative self-talk can amplify these emotions. By fostering a compassionate internal dialogue, veterans can respond to their hallucinations with kindness and understanding rather than self-blame, self-criticism, or panic. For example, instead of thinking, “I’m losing my mind” or “This is terrifying,” veterans can reframe their thoughts with statements like, “This is a symptom of my condition, and I’m not in danger” or “It’s okay to feel scared, but I can handle this.” This shift toward self-compassion can reduce anxiety, build resilience, and empower veterans to manage their CBS symptoms with greater emotional stability and a sense of control.

The following are some examples of compassionate self-talk that can help veterans feel more supported, calm, and empowered when experiencing a CBS visual hallucination:

1. **Normalising the Experience:**
   - "What I’m seeing is a symptom of my condition, and it doesn’t mean something is wrong with me."
   - "I’m not losing my mind; these hallucinations are part of CBS, and they don’t pose any danger."
2. **Offering Self-Kindness:**
   - "It’s okay to feel scared or confused right now. I’m doing my best, and that’s enough."
   - "I’ve been through a lot, and I give myself permission to be more patient and kinder."
3. **Reassuring with Self-Compassion:**
   - "These hallucinations don’t control me. I have the strength and resilience to handle them."
   - "It’s alright to feel overwhelmed, but I can take things one moment at a time and breathe through it."
4. **Promoting Resilience:**
   - "I have faced challenges before and know I can get through this, too."
   - "I’ve survived difficult times before, and I will manage this, just like I’ve managed other obstacles in my life."
5. **Encouraging Empowerment:**
   - "I can use the tools I’ve learned to stay grounded, calm, and in control."
   - "I can manage my thoughts and emotions, even when things feel out of control."
6. **Affirming Progress and Hope:**
   - "I am learning to cope better every day. It’s a journey, and I’m taking steps forward."
   - "There is support for me, and I don’t have to go through this alone."

By using compassionate self-talk, veterans can reduce the negative emotional impact of CBS, increase their sense of control, and enhance their overall resilience and well-being.

Identifying stress triggers that may lead to the onset of CBS visual hallucinations in military veterans is crucial for managing and reducing their frequency. One effective technique is to maintain a detailed symptom diary, where veterans can record the timing, context, and intensity of their hallucinations, noting any emotional or environmental factors that may have contributed. This could include changes in routine, stress from PTSD-related triggers, fatigue, or specific sensory inputs like bright lights or complex patterns. By recognising these patterns, veterans and healthcare providers can identify potential stressors and take preventive measures. Mindfulness practices are also beneficial, as they help veterans stay attuned to their internal states, enabling them to notice when stress is building and to implement coping strategies before hallucinations occur. Additionally, cognitive-behavioural approaches can be utilised to challenge and reframe the stressful thoughts that may trigger CBS symptoms, allowing veterans to manage their stress more effectively. Finally, encouraging regular social support and engagement in calming activities, such as relaxation exercises or hobbies, can help veterans lower overall stress levels and decrease the likelihood of CBS-related hallucinations.

Addressing and challenging the stigma and prejudice surrounding CBS among military veterans requires a multifaceted approach focusing on education, open dialogue, and normalising the experience. One key method is raising awareness about CBS, particularly within veteran communities and healthcare settings. This can be achieved by organising workshops, providing educational resources, and integrating CBS training into military health programs. It’s essential to emphasise that CBS is not a sign of mental illness but rather a visual symptom of vision loss, which helps veterans better understand the condition and reduces the shame often linked with it. Peer support groups can also play a pivotal role, allowing veterans to share their experiences and realise they are not alone, which can alleviate feelings of isolation. Encouraging open communication between veterans and healthcare providers regarding CBS and ensuring that veterans feel heard and supported can help minimise misunderstandings. Additionally, advocacy campaigns focused on CBS through veterans’ organisations or mental health groups can help challenge negative stereotypes and promote empathy, ultimately fostering a more supportive environment for veterans with the condition.

Practical triage and risk assessment are crucial for managing military veterans with CBS to ensure their symptoms are appropriately addressed and correctly diagnosed. Triage involves prioritising veterans based on the severity of their symptoms, especially when the emotional distress caused by CBS hallucinations is significant. Veterans experiencing heightened anxiety, confusion, or agitation due to CBS should be prioritised for immediate care, ensuring they receive emotional support and reassurance. A comprehensive risk assessment is essential to determine if the hallucinations are exacerbating underlying mental health conditions, such as PTSD, depression, or anxiety, which are prevalent in military populations. This assessment helps identify the level of care required, whether it’s brief support and psychoeducation or a more intensive, multidisciplinary approach involving mental health professionals specialising in trauma.

Improving access to evidence-based trauma treatments such as Trauma-Focused Cognitive Behavioural Therapy (TF-CBT) and Eye Movement Desensitization and Reprocessing (EMDR) is essential for providing veterans with CBS the tools they need to manage both the visual hallucinations and their emotional impacts. TF-CBT is particularly effective for veterans dealing with the emotional fallout from trauma and the distress caused by CBS, aiding them in reframing negative thought patterns related to the hallucinations and trauma. EMDR, on the other hand, is a well-established therapeutic approach for processing traumatic memories and can also assist veterans in coping with the psychological distress triggered by their CBS symptoms and their link with adverse life experiences: past, present, and future. Ensuring timely referrals to these therapies, along with proper triage and initial risk assessment, allows veterans to receive comprehensive, trauma-informed care, which is critical for improving their long-term mental health and quality of life. Additionally, integrating these therapies within veteran healthcare systems can enhance access to care, reduce waiting times, and increase the chances of successful treatment outcomes.

Enhancing awareness of CBS in public and healthcare systems across the lifespan, especially among military veterans, involves a multifaceted strategy focusing on education, training, and community engagement. First, integrating CBS education into veteran-focused healthcare programs is essential. This can include training healthcare professionals- ophthalmologists, psychologists, and primary care providers- to recognise CBS symptoms early, ensuring timely diagnosis and appropriate intervention. Public awareness campaigns should aim to educate the broader community about CBS, emphasising that it results from significant vision loss rather than being a psychiatric disorder. Collaborating with veterans' organisations and public health initiatives, media campaigns utilising print, radio, and digital platforms can help normalise CBS and reduce the stigma associated with visual hallucinations. Additionally, tailored educational materials for veterans can be distributed in clinics, military support groups, and veteran outreach programs to reach those most at risk.

Cultural sensitivity is a crucial aspect of enhancing awareness, as different veteran populations may have unique perceptions of CBS, shaped by their cultural backgrounds and past experiences. For instance, military veterans from diverse racial or ethnic groups may interpret hallucinations differently or may be more likely to experience stigma from both within their communities and from healthcare professionals. Therefore, culturally tailored training for healthcare providers is essential to ensure they are attuned to the unique needs of veterans from various cultural contexts. This includes understanding how cultural beliefs about mental health and vision loss may affect how veterans discuss and seek help for CBS. Additionally, outreach programs should be culturally appropriate, employing languages, symbols, and references familiar to the target audience to foster understanding and trust. This strategy will aid in the early detection and management of CBS and create a supportive environment where veterans feel comfortable seeking help without fear of judgment or misunderstanding.

[Brief Music Break here]

**Section 3: A Summary of what we have learnt so far**

In conclusion, raising awareness, challenging stigma, improving access to services, and fostering further research and development in CBS among the military veteran population are essential steps toward ensuring better mental health and quality of life for veterans affected by this condition. Many veterans experience significant vision loss and may be at higher risk for CBS, yet the syndrome remains under-recognized and misunderstood. By increasing awareness, we can empower veterans and healthcare professionals to recognise the symptoms early, reduce the psychological distress caused by hallucinations, and encourage more proactive management. Challenging the stigma surrounding CBS, particularly within military communities, will create an environment where veterans feel safe seeking help without fear of judgment or misdiagnosis. Furthermore, improving access to specialised services, including evidence-based trauma therapies, is crucial for providing veterans with the tools they need to cope effectively with CBS and its emotional impact. Finally, ongoing research and development into CBS will provide valuable insights into its causes, treatments, and interventions, ensuring that the unique needs of military veterans are met as effectively and compassionately as possible. Moving forward, we must continue prioritising these efforts to support veterans in managing CBS and improving their well-being.

As Mary, a military veteran, disclosed:

“I see a brown rabbit, clear as day, but I never told anybody, certainly not for at least the first six months. I was petrified that people would think that I was starting with dementia or going crazy.

Increasing awareness about CBS, challenging stigma, nurturing better self-care and management, and obtaining better access to appropriate help and support are essential for our military veterans with CBS to ensure that Mary gets the right help and support she needs.

For the military veteran with CBS, a reminder strategy for self-care:

**CLEAR**

**C** – stay **CALM**

**L** – **LOOK** away or blink

**E** – **ENGAGE** in a different activity

**A** – **ACKNOWLEDGE** the image is not real

**R** – **REASSURE** that you are safe

**CLEAR**

We hope you have enjoyed this podcast. If you remember just one key message from all that we have discussed, it will hopefully be this: ***‘You are not your Charles Bonnet Syndrome, for this visual hallucination will pass.”***

The Northern Hub for Veteran and Military Families’ Research at Northumbria University at Newcastle brought this podcast to you, funded by the Ministry of Defence and the Office of Veterans’ Affairs.

We want to extend our sincere gratitude to the dedicated team at Sight Scotland Veterans, whose support and collaboration made this work possible. And, of course, a very special thank you to all the military veterans who generously gave their time to us to share their experiences of CBS.

If you’d like further information about Charles Bonnet Syndrome, or if you’re seeking help and support, please visit the following websites: www.sightscotland.org.uk (Sight Scotland and Sight Scotland Veterans) www.charlesbonnetsyndrome.uk (Esme’s Umbrella) www.rnib.org.uk (Royal National Institute of Blind People) www.blindveterans.org.uk (Blind Veterans UK)

Podcast written by Dr Jemma McCready, Professor Derek Farrell MBE and Professor Renata Gomes**.** Audio delivered by Associate Professor Jane Arnfield**.** Podcast created by Senior Broadcast Technician Mike Booth

Insert final music piece here

# Podcast script and link for military veterans and their family members/ carers

**Link to podcast: …..**

[Intro jingle]

Hello and welcome to this podcast. I’m your host, Jane Arnfield, and today we’re going to discuss a condition that affects many military veterans living with vision loss: Charles Bonnet Syndrome, also pronounced Charles Bonnie for our Scottish listeners.

Over the next 20 minutes, we’ll explore what Charles Bonnet Syndrome is, why it happens, and what experiencing it can be like. We’ll also give some practical tips to help manage it, as well as discuss where you can go for further help and support.

So, if you’re experiencing vision changes due to conditions like, but not limited to, Macular Degeneration, Glaucoma, or Giant Cell Arteritis, or if you support someone who is, then this podcast is for you.

Let's start by talking about what Charles Bonnet Syndrome is and why it happens.

[Short transition jingle]

Charles Bonnet Syndrome, or CBS for short, occurs when individuals with either complete or partial vision loss start to experience visual hallucinations. These hallucinations happen even though the person is cognitively healthy and fully aware that what they’re seeing isn't real. It’s simply your brain's way of reacting when your eyes aren’t sending enough information to your brain. These visual hallucinations are not a sign of dementia.

You might be wondering, “Why would my brain create pictures if I’m not actually seeing them?” Well, our eyes and our brain are a team. Under normal circumstances, your eyes send detailed images to your brain. When those images get blurry or don’t come through at all due to a visual impairment, your brain, which is eager for input, sometimes makes up its own pictures. These aren’t memories or flashbacks like those experienced in post-traumatic stress disorder, they’re completely new images your brain creates to fill the gaps.

Think of it like a radio that isn’t receiving a proper signal – it sometimes fills the silence with static or unexpected sounds. Well, your brain does the same thing, filling the gaps with images. This ‘filling in’ process isn’t something to be frightened of. It’s simply a quirk of the way our brains work.

So now that we know what CBS is and why it happens, let’s discuss what kinds of things people with CBS see and what they experience.

[Short transition jingle]

So what kind of things do people with Charles Bonnet Syndrome see?

It’s worth mentioning early on that the experience of CBS can vary widely from person to person. Visual hallucinations can range from simple shapes or lights, like flashes or colourful patterns, to more complex and detailed images, like faces, animals, or even moving scenes that seem almost real. Some of the images can be pleasant, like visions of flowers, butterflies and rabbits, while others can be more startling like tigers, insects and bugs, or faces of gargoyles. Often these images appear without warning and can happen when watching TV, lying in bed, or even when walking outside.

We asked some military veterans at Sight Scotland Veterans, a charity supporting ex-military service personnel impacted by sight loss, to describe some of their experiences with CBS hallucinations. James, a retired navy officer, told us:

“*I was out walking my dog once and I could see two people approaching me. They were ladies, and they were dressed in Japanese kimono and they walked up to me until they were about 20, 30 feet away, and I could see the faces by that time, they were quite clear, and I just watched them, and then I said through my voice ‘Hello, can you see me?’, and they just vanished, they just vanished”.*

Another military veteran, Valerie, described seeing small creatures:

*“I saw mice running across the floor, had they of been real I wouldn't have seen them, I’d have been out the door, but I knew they weren’t real ‘cos all of a sudden they would disappear”.*

And Edith, a retired army nurse, also had similar experiences:

*“It's always the same rabbit that keeps appearing in my living room… he's a perfect brown, like a film star rabbit, he’s beautiful, he's got the loveliest face, and I know he's not real because he never chases anything”.*

Those kinds of hallucinations we’ve just heard about from James, Valerie and Edith, are considered complex and dynamic. However, not all hallucinations can occur like this. Some hallucinations might be stationary or static, those like the ones William, a retired RAF pilot, experiences:

*“Well, see when I got in my bed, I can see a big wall in front of me, lovely, lovely colours, lovely colours, and I says to my wife, ‘that's Charles Bonnet back again’”.*

What all these hallucinations have in common though, are that they are typically very detailed and realistic, and they can, at first, be hard to recognise as hallucinations due to how real they appear. Most veterans report that their hallucinations tend to last a few seconds, however sometimes they might last for a bit longer, like a couple of minutes or so. Some people also report having several hallucinations a day. Again, it’s all very unique to the veteran experiencing it; meaning no two experiences are exactly the same.

It’s worth remembering though, that these visual experiences are a normal response to vision loss, and that experiencing visual hallucinations does not mean you, or the person you care for, are losing their mind or showing signs of cognitive decline, for example signs of dementia.

We’ll explore practical techniques for managing hallucinations in a little bit. But for now, you might be wondering: what are the chances of getting CBS, and what sort of things might trigger hallucinations?

[Short transition jingle]

So, how common is Charles Bonnet Syndrome and what triggers a hallucination episode?

Studies suggest that around 1 in 5 people with vision loss may experience visual hallucinations. It tends to affect people between ages 70 to 85, however, it can occur at any age, especially if the visual impairment affects both eyes. However, experts believe that CBS might be under reported. For instance, some individuals might keep their experiences to themselves and not disclose their symptoms and experiences to family members or healthcare professionals, for fear of what others may think, or do, in response to the information. Also, healthcare professionals who aren’t familiar with CBS might mistakenly attribute hallucinations to cognitive impairments, like dementia, or conditions such as post-traumatic stress disorder, potentially leading to misdiagnosis.

With regards to triggers, it’s thought that both over and under excitability of the brain can increase the likelihood of experiencing visual hallucinations. For example, it’s common for hallucinations to be triggered by sudden changes in lighting, such as going from a brightly lit area into a darker space, or vice versa. This is because the brain tries to adjust to the shifting of visual conditions.

Our research with military veterans shows that stress and tiredness trigger visual hallucinations, in addition to other factors such as loneliness or isolation. It’s thought that during these times, the brain becomes more sensitive to changes in visual input, which triggers hallucinations.

It’s important to keep in mind that the triggers we’ve discussed aren’t exhaustive and there may be other factors that can trigger visual hallucinations, which can vary from person to person.

Now that we've discussed some of the factors that could potentially trigger hallucinations, let's explore some practical strategies that can help manage these visual experiences, should they occur.

[Short transition jingle]

What do you do if you notice visual hallucinations?

At the moment, there’s no definitive cure for CBS. Some medications, such as antipsychotics, anticonvulsants, or antidepressants have been tested with the aim of reducing or stopping visual hallucinations but have mostly been found to be ineffective.

Because of this, practical self-help strategies have become the main way to help manage visual hallucinations. One of the most recommended and effective strategies reported by others experiencing CBS is the distraction method.

This involves simple actions such as blinking rapidly, closing your eyes briefly, or moving your eyes and head around, so looking up, down, or side to side. Some people even choose to gently interact with the hallucination by speaking out loud to it, reaching out with their hand, or moving towards it, as this can help to dispel the vision. If those techniques don’t work, you could also try moving out of the room or area, or even changing the lighting levels.

Research shows that about 70% of people who experience visual hallucinations, find these distraction methods effective for reducing or even completely stopping their visions. Experts believe this happens because the distraction changes your attention to a new task or sensation, which refocuses the brain and interrupts the hallucination.

Another helpful strategy is to identify your own triggers and address them directly. For example, if you notice that hallucinations increase during times of stress, then you may find regularly engaging in relaxation activities like listening to your favourite music, or an audiobook, such as those offered for free by the Royal National Institute of Blind People, or even engaging with some meditation or relaxation podcasts, might be helpful for managing stress and reducing the likelihood of experiencing a visual hallucination.

Similarly, if tiredness is one of your triggers, you might find it beneficial to take brief naps or rest your eyes at several points throughout the day. It might also be helpful to pace yourself throughout your daily activities to avoid overdoing it, so you can maintain your energy levels to try and avoid triggering a visual hallucination.

It's also completely understandable to have an emotional reaction to the visual hallucinations, but the way we talk to ourselves in these moments can make a big difference. Instead of thinking, 'Something is wrong with me' or 'This is terrifying,' try to reframe those thoughts with other thoughts like, 'This is just a symptom of my vision loss, I'm not in danger' or 'It's okay to feel startled, but I can manage this.' Shifting to a more understanding and compassionate mindset can help reduce anxiety and give you a greater sense of control over the experience. Remember, CBS is not a sign of mental illness, it's simply your brain adapting to changes in vision.

Not all of these strategies will work for everyone, and it might take a little time to find something that works for you, but it’s important to keep trying. If you experience a visual hallucination, there’s a simple word I’d like you to remember: CLEAR [spell word out] C.L.E.A.R.

C is for Calm, take deep breaths – Take a slow, deep breath and stay calm

L is for Look away or blink – Try blinking or shifting your gaze

E is for Engage – Engage in a different activity

A is for Acknowledge – Acknowledge the images aren’t real

R is for Reassure - Reassure yourself that you're safe and that the image will pass

Now that we know what to do to help clear a visual hallucination, let’s talk about what you need to do next and where to go for additional help and support.

[Short transition jingle]

So what next, where can you go for help and support?

Firstly, if you or someone you know is experiencing visual hallucinations, it's important to speak to your eye healthcare provider and your GP as soon as possible. They can help you rule out any other causes and confirm whether CBS could be the reason behind the visual hallucinations.

It’s completely understandable to feel anxious, confused or even a bit frightened when visual hallucinations first occur. While some people might find these visions pleasing or intriguing, others can find them unsettling. For most people, simply understanding what’s happening and why, is enough to ease worries, but it's always best to speak openly about your experiences with a healthcare professional, family member, or someone else you trust if you're feeling distressed.

If your hallucinations regularly affect your ability to move safely, for example you’re seeing objects blocking your path when walking, crossing roads, or descending stairs, then you should discuss this promptly with your GP. These visual experiences could affect your confidence in getting around safely and may even increase your risk of falls. Your GP can offer specific advice to help you stay mobile and safe.

Next, regular appointments with your eye healthcare provider can also help. Ensuring your eye prescriptions are up to date can help manage visual symptoms by promoting, where possible, clearer, more comfortable vision overall.

Finally, connecting with support groups and sight-loss charities can be particularly valuable. Charities like Sight Scotland, Sight Scotland Veterans the Royal National Institute of Blind People (RNIB), Blind Veterans UK, and Esme’s Umbrella can offer support, reassurance, and practical advice for living with and managing CBS. Most of these charities also offer helplines where you can speak with someone who can provide more tailored information and guidance on CBS and can support you further with what to do next.

[Short transition jingle]

So today, we've explored what Charles Bonnet Syndrome is, why it happens, and how it might affect veterans living with vision loss. We’ve heard from military veterans who described a variety of visual hallucinations, from simple lights and patterns to more detailed visions like animals or people.

We also discussed practical strategies to manage visual hallucinations, and introduced the acronym CLEAR, to help remember what to do if you experience a visual hallucination. So, remember C – Calm, take deep breaths; L – Look away or blink; E – Engage in a different activity; A – Acknowledge the images aren’t real; and R – Reassure yourself that you’re safe. C – CALM; L – LOOK AWAY; E – ENGAGE IN A DIFFERENT ACTIVITY; A –ACKNOWLEDGE; R – REASSURE.

Finally, remember to reach out if you or someone you support experiences visual hallucinations. Talk openly to your GP or eye healthcare provider, and stay up to date with regular eye check-ups. Connecting with sight-loss charities and support groups can also offer further support and practical guidance on what to do next.

Thanks for listening. Take care and remember to keep things CLEAR.

[Short transition jingle]

This podcast was brought to you by the Northern Hub for Veteran and Military Families’ Research at Northumbria University at Newcastle, funded by the Ministry of Defence and the Office of Veterans’ Affairs.

We would like to extend our sincere gratitude to the dedicated team at Sight Scotland Veterans, whose support and collaboration made this work possible. And of course, a very special thank you to all of the military veterans who generously gave their time to share their experiences of CBS.

If you’d like further information about Charles Bonnet Syndrome, or if you’re seeking help and support, please visit the websites of the following charities: Sight Scotland and Sight Scotland Veterans, Esme’s Umbrella, Royal National Institute of Blind People and Blind Veterans UK. You can also call 0800 035 6409, to speak to a member of the Sight Scotland Veterans team.

Podcast written by Dr Jemma McCready, Professor Derek Farrell MBE and Professor Renata Gomes. Audio delivered by Associate Professor Jane Arnfield. Podcast created by Senior Broadcast Technician Mike Booth

[Outro jingle]

#
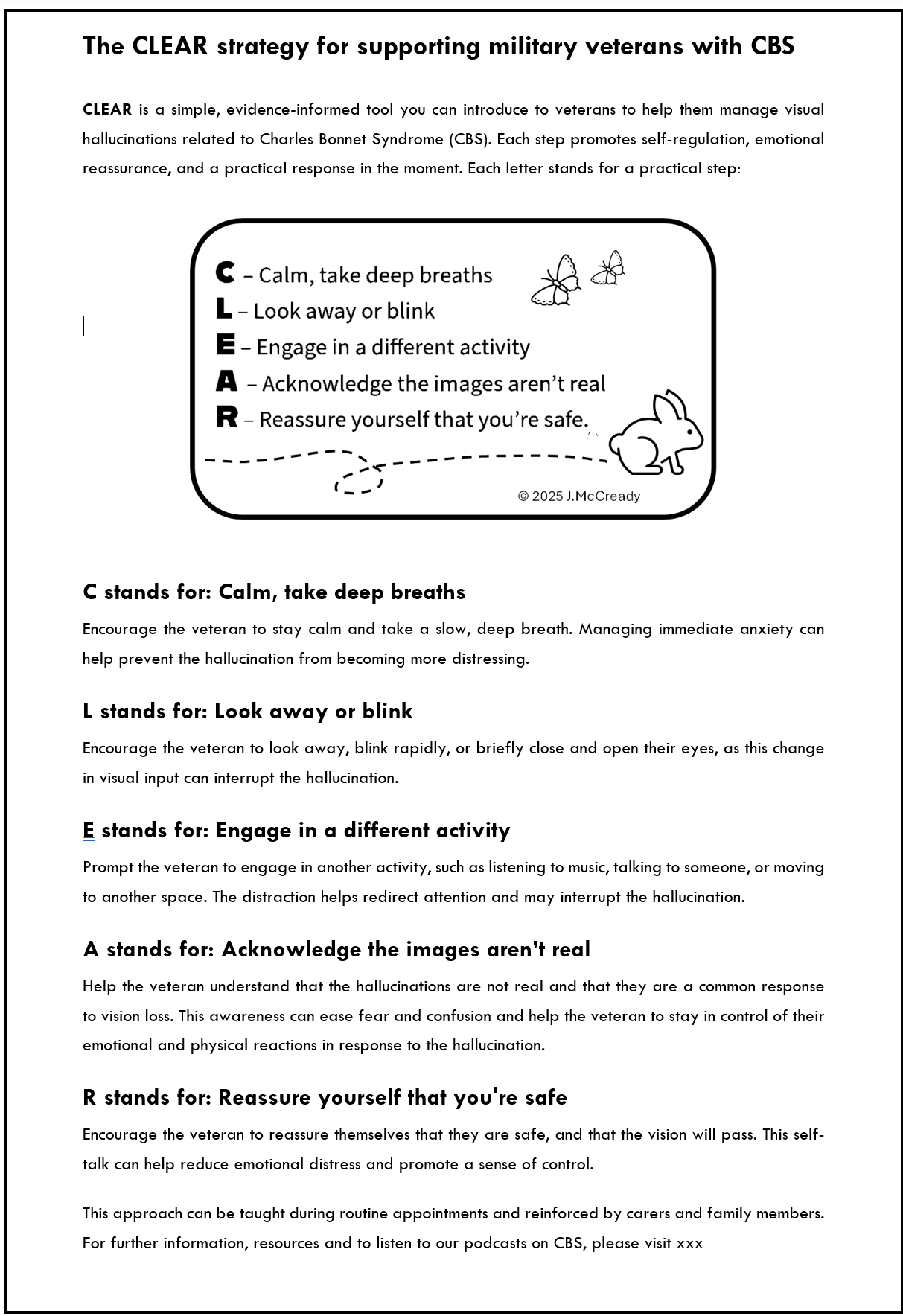
Guidance on implementing CLEAR strategy for healthcare professionals

#
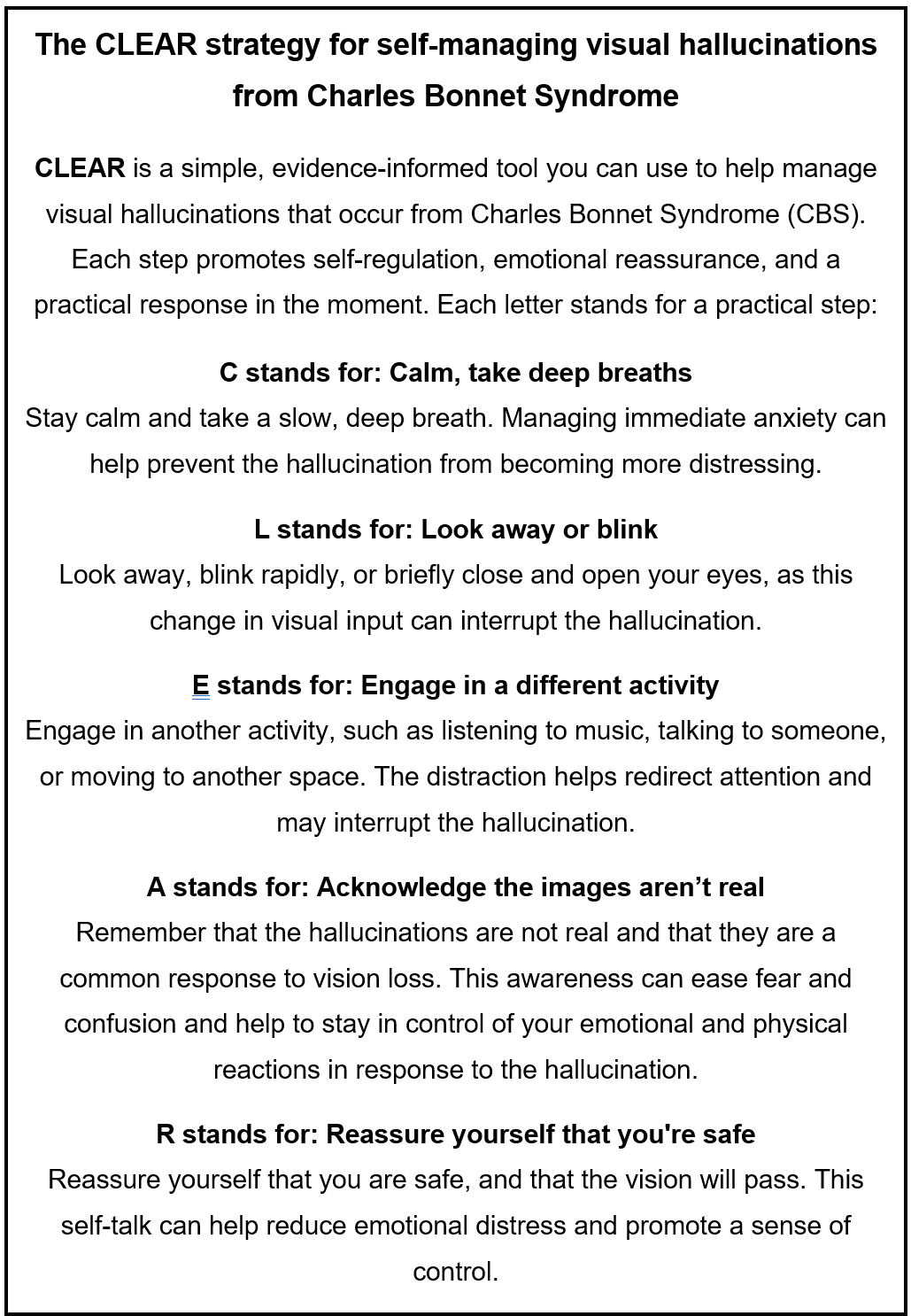
Guidance on implementing CLEAR strategy for military veterans and their family members/ carers


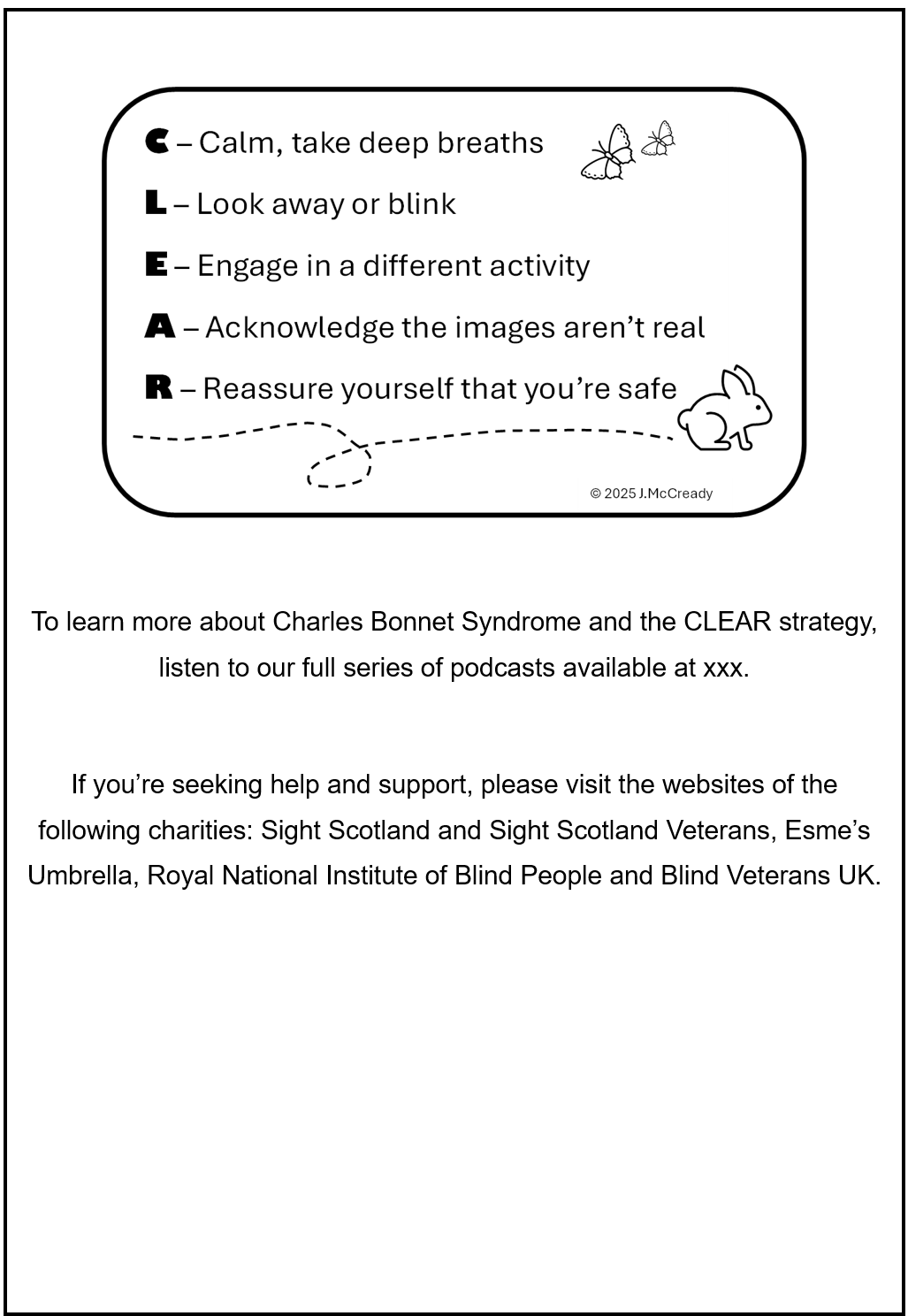


# Short podcast script and link for military veterans and their family members/ carers

Link to podcast: ……….

[Intro jingle]

Hello and welcome. In this podcast, we’re going to discuss a condition that affects many military veterans living with vision loss: Charles Bonnet Syndrome. Over the next few minutes, we’ll explore what Charles Bonnet Syndrome is, why it happens, what it can feel like, and what you can do if it happens to you. We’ll also let you know who to turn to for support and advice. So, if you’re living with a visual condition like Macular Degeneration, Glaucoma, or Giant Cell Arteritis, then this podcast is for you.

[Short transition jingle]

So, let’s begin with the basics. What exactly is Charles Bonnet Syndrome?

Charles Bonnet Syndrome or CBS for short, is a condition where people with vision loss start to see things that aren’t really there – these are called visual hallucinations. These hallucinations happen even though the person is cognitively healthy and fully aware that what they’re seeing isn't real. You might be wondering, “Why would my brain create pictures if I’m not actually seeing them?” Well, our eyes and our brain are a team. Under normal circumstances, your eyes send detailed images to your brain. When those images get blurry or don’t come through at all due to a visual impairment, your brain, which is eager for input, sometimes makes up its own pictures. These aren’t memories or flashbacks like those experienced in post-traumatic stress disorder, they’re completely new images your brain creates to fill the gaps.

And CBS is more common than you might think—it's estimated that around 1 in 5 people with sight loss experience it. It’s most often seen in people aged 70 to 85, but it can happen at any age, particularly when vision loss affects both eyes.

[Short transition jingle]

So, what kind of things might someone with CBS actually see?

Well, visual hallucinations can vary a lot from person to person. Some veterans have reported seeing simple shapes, flashes of lights, or colourful patterns. While others see more detailed images like animals, people, or even full scenes such as buildings or landscapes.

Some of these images can be pleasant, like flowers, rabbits or butterflies. Others might be a bit startling such as insects, tigers or faces of gargoyles. These images can appear suddenly and might last just a few seconds or a few minutes. Some veterans report seeing them now and then, while others see them more often throughout the day.

The content and frequency of hallucinations is different for everyone. But one thing is important to remember – it’s a normal reaction to sight loss, not a sign of dementia or mental illness.

[Short transition jingle]

You might now be asking, “What causes these hallucinations to happen?”

Well, several things can trigger a visual hallucination. For example, sudden changes in lighting, like going from a brightly lit area into a darker space or vice versa can set them off. So too can stress and tiredness or even feeling lonely or isolated. It’s thought that during these times, the brain becomes more sensitive to changes in visual input and that can lead to visual hallucinations.

Not everyone has the same triggers, so it’s helpful to notice when your hallucinations tend to happen.

[Short transition jingle]

So, what can you do if you experience a visual hallucination?

Here’s a simple way to remember what to do. Think of the word CLEAR - C.L.E.A.R.

C is for Calm – Take a slow, deep breath and stay calm

L is for Look away – Try blinking or shifting your gaze

E is for Engage – Engage in something else to distract your mind

A is for Acknowledge – Acknowledge that these images aren’t real

R is for Reassure - Reassure yourself that you're safe and that the image will pass

This can help you to feel more in control and less anxious in the moment.

[Short transition jingle]

Once you’ve recognised the signs of CBS, it’s important to talk about it.

Speak to your eye healthcare provider and your GP as soon as possible. They can help rule out any other causes and confirm whether CBS could be the reason behind the visual hallucinations.

You might also want to talk to a family member or friend. Sharing what you’re experiencing can help others understand and support you better. Feel free to share this podcast with them too.

And finally, connect with support groups and sight-loss charities. Organisations like Sight Scotland Veterans, the Royal National Institute of Blind People, Blind Veterans UK, and Esme’s Umbrella offer helpful resources, support and practical guidance on CBS and can help you with what to do next.

[Short transition jingle]

To wrap up – CBS can be surprising, and at times unsettling, but remember, it’s your brains natural response to vision loss, and you’re not alone in experiencing it. By understanding what it is, noticing what triggers it, and using the CLEAR technique, you can manage these experiences calmly and confidently.

So, remember: C – Calm, take deep breaths; L – Look away or blink; E – Engage in a different activity; A – Acknowledge the images aren’t real; and R – Reassure yourself that you’re safe.

[Short transition jingle]

This podcast was brought to you by the Northern Hub for Veteran and Military Families’ Research at Northumbria University at Newcastle, funded by the Ministry of Defence and the Office of Veterans’ Affairs.

A special thank you to Sight Scotland Veterans and to all the veterans who kindly shared their stories.

Thanks for listening. Take care and remember to keep things CLEAR.

Podcast written by Dr Jemma McCready, Professor Derek Farrell MBE and Professor Renata Gomes**.** Audio delivered by Associate Professor Jane Arnfield**.** Podcast created by Senior Broadcast Technician Mike Booth

[Outro jingle]

# Animated video for general audiences

**Link to animated video: ……**

1. Hedges (2006) [↑](#footnote-ref-1)
2. Bonnet (1760) [↑](#footnote-ref-2)
3. de Morsier (1967) [↑](#footnote-ref-3)
4. Subhi et al., (2022) [↑](#footnote-ref-4)
5. Christoph et al., (2024) [↑](#footnote-ref-5)
6. Tan et al., (2004) [↑](#footnote-ref-6)
7. Jones et al., (2025) [↑](#footnote-ref-7)
8. Dave et al., (2024) [↑](#footnote-ref-8)
